# PARP-1 as a novel target in endocrine-resistant breast cancer

**DOI:** 10.1186/s13046-025-03441-4

**Published:** 2025-06-16

**Authors:** Azzurra Zicarelli, Marianna Talia, Muriel Lainé, Rosamaria Lappano, Marcello Maggiolini, Geoffrey L. Greene

**Affiliations:** 1 Ben May Department for Cancer Research, University of Chicago, Chicago, IL USA; 2 Department of Medicine and Surgery, University of Enna “Kore”, Enna, 94100 Italy; 3 Department of Pharmacy, Health and Nutritional Sciences, University of Calabria, Rende, 87036 Italy

**Keywords:** Breast cancer, Endocrine therapy, PARP-1, Niraparib

## Abstract

**Background:**

Several mechanisms are involved in the resistance to endocrine therapy (ET) in estrogen receptor (ERα)-positive breast cancer (BC), including acquired mutations of ERα gene (ESR1). For example, the frequent mutation, Y537S, was shown to trigger a constitutively active receptor leading to reduced affinity for both agonist and antagonist ligands. The development of more comprehensive therapies remains a challenge in BC patients exhibiting activating mutations in ERα. Here, we show that Poly (ADP-ribose) polymerase-1 (PARP-1) may be considered as a novel therapeutic target in ERα-positive BC.

**Methods:**

ERα wild type or Y537S mutated MCF7 and T47D BC cell lines were used as model systems. Immunoblotting, immunofluorescence, gene silencing, real-time PCR, promoter assays, chromatin immunoprecipitation sequencing (ChIP-seq) as well as cell viability, colony and cell cycle assays served to investigate the involvement of PARP-1 in BC progression. The growth of MCF7 ERα Y537S cells injected into the mammary ducts of NSG mice and treated with the ERα antagonist lasofoxifene or the PARP-1 inhibitor niraparib was monitored by luminescence imaging, weight measurement, and histological analysis. RNA sequencing studies were performed on the above-described xenograft tumors. METABRIC dataset was used to evaluate the clinical significance of PARP-1 and the biological role of the PARP-1-associated genes in ERα-positive BC patients.

**Results:**

We first demonstrated that the up-regulation of PARP-1 expression induced by estrogens is abrogated either by inhibiting or silencing ERα in MCF7 and T47D BC cells expressing ERα wild type or Y537S mutation. We then showed that PARP-1 is involved in the binding of ERα and its co-activator FoxA1 to the promoters of several target genes, as determined by ChIP-sequencing studies. Of note, the inhibition of PARP-1 prevented the proliferative effects mediated by ERα in BC cells expressing either wild type or Y537S ERα. In accordance with these findings, the growth of xenograft tumors derived from MCF7 ERα Y537S BC cells was significantly reduced using niraparib and lasofoxifene. Finally, RNA-sequencing analyses showed that ERα signaling is downregulated by niraparib compared to vehicle-treated tumors.

**Conclusions:**

Overall, our results suggest that PARP-1 should be explored as a potential target in comprehensive therapeutic approaches in ET-resistant BC.

**Supplementary Information:**

The online version contains supplementary material available at 10.1186/s13046-025-03441-4.

## Background

Breast cancer (BC) is the most frequently diagnosed malignancy of epithelial origin in women worldwide, and it represents the second cause of cancer death [1–3]. Estrogens are a substantial risk factor for BC progression due to their stimulatory effects on the proliferation of both normal and neoplastic breast epithelium cells [4, 5]. The biological effects of estrogens are primarily mediated by the estrogen receptors ERα and ERβ, which are encoded by the ESR1 and ESR2 genes, respectively [6, 7]. ERα is the primary receptor involved in the development and progression of ERα-positive BC because it activates signaling pathways that promote cell growth, survival, invasion and metastasis [8]. Approximately 80% of BCs are ERα-positive and endocrine therapy (ET) remains the first line adjuvant treatment [9, 10]. ET has significantly improved the outcomes of ERα-positive BC patients [11]. Specifically, selective ER modulators (SERMs, i.e. tamoxifen), selective estrogen receptor degraders (SERDs, i.e. fulvestrant), and aromatase inhibitors (AIs, i.e. letrozole) have been approved and largely used as adjuvant treatments in ERα-positive BC patients [12]. Unfortunately, approximately 40% of patients develop resistance to ET [13–15]. To date, several molecular mechanisms have been implicated in ET resistance, including acquired ESR1 mutations [16]. The two most common somatic ESR1 mutations are Y537S and D538G, located at the N-terminus of helix 12 (H12) in the ligand binding domain (LBD), which is a critical structural regulator of ERα coactivator recruitment [17]. Regarding the constitutively active ESR1 Y537S mutation, its ability to stabilize the activating function-2 (AF-2) domain of the LBD in the agonist-bound conformation may interfere with the capability of SERMs and SERDs to inhibit ERα [18]. In addition, other mechanisms have been implicated in ERα Y537S-mediated resistance to ET and BC progression [17–19].

Poly (ADP-ribose) polymerase-1 (PARP-1) has recently gained considerable attention as a therapeutic target in BC [20]. PARP-1 is involved in the ADP-ribosyl transferase of many known substrates [21, 22] as well as in triggering the DNA damage response by transferring poly-ADP-ribose molecules to target proteins [23, 24]. Recent studies have also identified novel PARP-1 interaction partners modulating its enzymatic activity [25]. To date, PARP inhibitors have been used even in combination therapies with other molecules for the treatment of locally advanced or metastatic BC [26]. Using ERα-positive MCF7 and T47D BC cells that were engineered to express the Y537S mutation, we demonstrate that a functional crosstalk occurs between ERα and PARP-1 in both ERα wild type and Y537S-mutated BC cells toward transcriptional and growth responses. Notably, the inhibition of PARP-1 reduced ERα-mediated proliferation of the BC cells used. Overall, our results suggest that PARP-1 should be considered as a novel target for more comprehensive treatments of BC expressing either ERα wild type or Y537S mutated ERα.

## Methods

### Cell lines and growth conditions

ERα-positive BC MCF7 and T47D cells, originally obtained from ATCC (American Type Culture Collection), were engineered using adeno-associated virus recombinant viral vectors to express ESR1 mutation, ERα Y537S. MCF7 ERα wild type cells and MCF7 ERα Y537S cells were generated and gifted by Ben Ho Park, originally at Johns Hopkins University and now at Vanderbilt University [27]. T47D ERα wild type cells and T47D ERα Y537S cells were generated and gifted by Steffi Oesterreich at the University of Pittsburgh [27]. MCF7 ERα wild type cells were cultured in phenol red-free Dulbecco’s Modified Eagle Medium (DMEM) containing 5% fetal bovine serum (FBS), 1% Penicillin/Streptomycin (Pen/Strep), and 1% L-Glutamine (L-Glu). MCF7 ERα Y537S cells were maintained in phenol red-free DMEM containing 10% charcoal-stripped serum (CSS), 1% Pen/Strep and 1% L-Glu. T47D ERα wild type cells were cultured in phenol red-free Roswell Park Memorial Institute 1640 (RPMI) media containing 10% FBS, 1% Pen/Strep, and 1% L-Glu. T47D ERα Y537S cells were maintained in phenol red-free RPMI media containing 10% CSS, 1% Pen/Strep, and 1% L-Glu. Cells were cultured in serum free medium 24 h before experiments. All cell lines were validated for ERα receptor status (wild type or Y537S mutated) through next-generation sequencing (NGS) completed by the University of Illinois at Chicago Genome Research Core. Cells were tested for mycoplasma after thawing fresh cells and before beginning experimentation. Testing was completed using the MycoAlert Mycoplasma Detection Kit (Lonza Bioscience #LT07-318).

### Animal studies

Mouse studies complied with an approved Institutional Animal Care and Use Committee protocol at the University of Chicago. MCF7 ERα Y537S cells underwent transduction with a lentiviral vector (pFU-Luc2-eGFP) containing a fusion gene of luciferase and green fluorescent protein (GFP), driven by a ubiquitin promoter at a multiplicity of infection (MOI) of 5 while in suspension, followed by plating [28]. Subsequently, cells were subjected to genotyping before murine injection. DNA extraction was performed using the DNeasy Blood and Tissue Kit (Cat #69504, Qiagen), with the verification of ERα mutation accomplished via sequencing utilizing CCCCTTCTAGGGATTTCAGC as the sequencing primer. To better mimic the progression of ductal lesions to invasive disease, a mammary intraductal (MIND) mouse model [29, 30] was used. NOD.CB17-Prkdcscid/J mice were anesthetized by intraperitoneal injections with a ketamine/xylazine (100/5 mg/kg) mixture in Phosphate-Buffered Saline (PBS) before cancer cell injections. As previously performed, single-cell suspensions of 2.5 × 10^5^ MCF7 ERα Y537S cells were injected into the mammary ducts of inguinal glands [28, 29]. Tumor cell growth in situ was followed once weekly or biweekly after injection via luminescence imaging with LagoX (Spectral Instruments). Before imaging, mice were injected with 100µL of 0.1 M luciferin (Cat #122799, Perkin Elmer XenoLight) dissolved in PBS.

### Compounds and antibodies

Vehicle (ethanol) was used as a control. For in vitro assays, cells were treated with 10 nM E_2_ (Cat #E2758, Sigma), 1µM niraparib (Cat #HY-10619, MedChemExpress), 1µM fulvestrant (Cat #HY-13636, MedChemExpress), 1µM olaparib (Cat #HY-10162, MedChemExpress), and lasofoxifene (Cat #SML1026, Merck Life Science). For in vivo experiments, lasofoxifene at 5 mg/kg in 100 µM of PBS containing 15% PEG 400 and niraparib at 10 mg/mouse in 100µL of mineral oil was administered 5 days/week via subcutaneous (s.c.) injection. Mice were sacrificed after 90 days of MCF7 ERα Y537S cells injection, and mammary gland tumors were weighed. PARP-1 rabbit polyclonal antiserum generated in the Kraus laboratory by using an antigen comprising the amino-terminal half of human PARP-1 (Cat #39559, Active Motif) was used (1:200) for PARP-1 detection in immunofluorescence assay [31]. PARP-1 rabbit polyclonal (1:200) and ERα (F10) mouse monoclonal (1:50) antibody (#sc-8002, Santa Cruz Biotechnology) were used for immunoblotting detection of PARP-1 and ERα, respectively. The following antibodies were used for ChIP and Re-Chip assays: FoxA1 rabbit polyclonal (#Ab23738 Abcam), ERα rabbit polyclonal (Cat#13-2012, EpiCypher) antibody, and Sp1 mouse monoclonal (#sc-420, Santa Cruz Biotechnology). Anti-pan-cytokeratin rabbit monoclonal antibody (Cat# ab234297, Abcam) was used in Immunohistochemical (IHC) staining. Primary, excised glands were stained using the anti-Ki67 antibody (Cat #RM-9106-s, Clone SP6, ThermoScientific).

### Histological analysis

Tissues were harvested and fixed in formalin. After scarification, hematoxylin and eosin (H&E), and IHC staining were performed on the mice glands. Histological analysis was conducted by the Human Tissue Resource Center (HRTC) at the University of Chicago. The slides were stained on Leica Bond RX Automatic Stainer. After Dewax, rehydration and antigen retrieval treatment (epitope retrieval solution II, AR9640, Leica Biosystems) for 20 min, anti-pan-cytokeratin antibody (1:2000) was applied on tissue sections for 60 min incubation and the antigen-antibody binding were detected with Bond Polymer Refine Detection kit without Post Primary (DS9800, Leica Biosystems). After deparaffinization and rehydration, tissue sections were treated with Target Retrieval Solution (Leica Biosystems, AR9640) for heat treatment for 20 min. The antigen-antibody binding was detected by ImmPress anti-mouse IgG Polymer-HRP kit (Vector Laboratories, MP-7402) and DAB (Leica Biosystems, Bond Polymer Refine kit). A mouse-on-mouse blocking kit (Vector Laboratories, MKB-2213) was used to block non-specific binding. The tissue sections were counterstained with hematoxylin and covered with cover glasses. Nikon Eclipse Ti2 microscope with 20× objective obtained high-resolution images. The samples were analyzed using the QuPath version 0.5.1 [32]. Results were plotted as histogram +/− SEM or using a boxplot.

### Gene silencing experiments

Dharmacon™ ON-TARGETplus Control non-targeting pool (Cat #D-001810-10-05, Horizon Discovery), PARP-1 SMARTpool (Cat #L-006656-03-0005, Horizon Discovery), ESR1 SMARTpool (Cat #L-003401-00-0005, Horizon Discovery and esiRNA Human PARP-2 (Cat #EHU025371, Merck Life Science) were used for silencing experiments. Cells were transfected using Lipofectamine™ RNAiMAX (Thermo Fisher Scientific) in OptiMEM (Gibco). After 24 h, all cell lines were exposed to treatments.

### RNA extraction

Cells were plated at 2 × 10^5^ cells/well. After reaching the optimum confluence, all cell lines were transferred into hormone-deprived medium. After 48 h, cells were treated for 1 h with 1µM fulvestrant or 1µM niraparib and then treated for 18 h with vehicle or 10 nM E_2_. RNA was extracted using the RNeasy Plus kit (Cat #74104, Qiagen) according to the manufacturer’s protocol. RNA concentrations were quantified using nanodrop nucleic acid measurement. Quantitative reverse transcription polymerase chain reaction (RT-qPCR) was used to quantify RNA expression. cDNA was synthesized from 1 µg RNA using 5X qScript Mastermix (Cat #95048, Quanta Bio) according to the Quanta Bio qScript protocol. For RNA-seq studies, the total RNA was extracted from mouse NOD SCID mammary glands using an RNA isolation kit (RNeasy@ Mini Kit, Qiagen). The quantity and quality of RNA were evaluated by Nanodrop and by running on agarose gel electrophoresis. RNA library preparation and sequencing were conducted at Azenta Life Sciences (South Plainfield, NJ, USA, https://www.azenta.com/) with Library Preparation with PolyA selection and Illumina Sequencing.

### RNA-sequencing pipeline

Total RNA samples were quantified using Qubit 3.0 Fluorometer (Life Technologies, Carlsbad, CA, USA) and RNA integrity was checked using Agilent TapeStation 4200 (Agilent Technologies, Palo Alto, CA, USA). ERCC Ex-fold RNA reagent (Cat #4456739, ThermoFisher Scientific) was added to normalized total RNA prior to library preparation following manufacturer’s protocol. RNA sequencing (RNA-seq) libraries were prepared using the NEBNext Ultra II RNA Library Prep Kit for Illumina using manufacturer’s instructions (NEB, Ipswich, MA, USA). Briefly, mRNAs were initially enriched with Oligod(T) beads. Enriched mRNAs were fragmented for 15 min at 94 °C. First strand and second strand cDNA were subsequently synthesized. cDNA fragments were end repaired and adenylated at 3’ends, and universal adapters were ligated to cDNA fragments, followed by index addition and library enrichment by PCR with limited cycles. The sequencing library was validated on the Agilent TapeStation (Agilent Technologies, Palo Alto, CA, USA), and quantified by using Qubit 2.0 Fluorometer (Invitrogen, Carlsbad, CA) as well as by quantitative PCR (KAPA Biosystems, Wilmington, MA, USA). The sequencing libraries were multiplexed and clustered onto a flowcell on the Illumina NovaSeq instrument according to manufacturer’s instructions. The samples were sequenced using a 2 × 150 bp Paired End (PE) configuration. Image analysis and base calling were conducted by the NovaSeq Control Software (NCS). Raw sequence data (.bcl files) generated from Illumina NovaSeq was converted into fastq files and de-multiplexed using Illumina bcl2fastq 2.20 software. One mismatch was allowed for index sequence identification. After investigating the quality of the raw data, sequence reads were trimmed to remove possible adapter sequences and nucleotides with poor quality. The trimmed reads were mapped to the reference genome available on ENSEMBL using the STAR aligner v.2.5.2b. The STAR aligner is a splice aligner that detects splice junctions and incorporates them to help align the entire read sequences. BAM files were generated as a result of this step. Unique gene hit counts were calculated by using feature Counts from the Subread package v.1.5.2. Only unique reads that fell within exon regions were counted. After the extraction of gene hit counts, the gene hit counts table was used for downstream differential expression analysis. Using DESeq2, a comparison of gene expression between the groups of samples was performed. The Wald test was used to generate p-values and Log2 fold changes. Genes with adjusted p-values < 0.05 and log2 fold changes ≤ -0.5 or ≥ 0.5 were called as differentially expressed genes (DEGs) for each comparison. Thereafter, the ReactomePA package [33] was used to group the differentially expressed genes in pathways, setting the following parameters: organism = “human”, p-value cut-off = 0.05. Gene expression data are available through NCBI Gene Expression Omnibus (GSE277883).

### Gene expression studies

Applied Biosystems™ TaqMan™ Fast Advanced Master Mix (Cat #4444557, ThermoFisher Scientific) and Human Beta-2-Microglobulin (B2M) endogenous control (Cat #4326319E, ThermoFisher Scientific) were used for RT-qPCR using a Roche Step-One Real-Time PCR machine (ThermoFisher Scientific). Gene specific primers (PARP-1, PARP-2, PGR, MYC, FOS, CCND1, E2F1, GREB1, TFF1, PDZK1) were purchased by Integrated DNA Technologies - IDT. Assays were performed in triplicate, and the results were normalized for B2M expression. Then, the results were calculated as fold induction of gene RNA expression normalized to B2M.

### Transfection and gene reporter assays

Cells (1 × 10^5^) were plated into 24-well plates in regular growth medium the day before transfection. Cell media were replaced with medium supplemented with 1% CSS and transfection was performed by using the FuGENE^®^ 4 K Transfection Reagent, as recommended by the manufacturer (Cat #E5911, Promega). Firefly luciferase reporter plasmids used were ERE-luc for ERα and GK1 [34, 35] for the Gal4 (GalERα) fusion protein [34]. The Renilla reniformis luciferase expression vector pRL-CMV (Promega) was used as internal transfection control. After 18 h, cells were treated with 10nM E_2_ in the presence or absence of 1µM fulvestrant or 1µM niraparib for additional 12 h. According to the manufacturer’s recommendations, luciferase activity was measured using the Dual-Glo^®^ Luciferase Kit (Cat #E2920, Promega). Firefly luciferase activity was normalized to the internal transfection control. Normalized relative light unit values obtained from cells treated with vehicle were set as one-fold induction, upon which the activity induced by treatments was calculated.

### Immunofluorescence microscopy

Cells were grown on a coverslip; next, they were fixed in iced methanol for 7 min, permeabilized with 0.2% Triton X-100, washed 3 times with PBS, and incubated at 4 °C overnight with the primary antibody. After incubation, cells were extensively washed with PBS, probed with Alexa Fluor 488 goat anti-rabbit IgG (1:250, Thermo Fisher Scientific) and 4′,6-diamidino-2-phenylindole dihydrochloride (DAPI) (1:1000, Merck Life Science). The images were captured using Nikon ECLIPSE Ti2 microscope with a 60 × objective and analyzed with the ImageJ software.

### Simple western (wes by protein simple)

Cells were lysed using M-PER™ Mammalian Protein Extraction Reagent (Cat #78501, Thermo Fisher Scientific) containing cOmplete™ EDTA-free Protease Inhibitor Cocktail (Pics, Cat #04693159001, Roche). Protein concentrations were quantified using the A280 Nanodrop program. All immunoblot detection using the Bio-Techne ProteinSimple detection reagents and 12–230 kDa Separation Module (Bio-Techne #SM-W001 and #DM-002) [36]. The experiments were conducted under reducing conditions (40 mM DTT). Separation took place at 375 V for 25 min. Afterward, the proteins were immobilized on the capillary wall, covalently labeled with biotin for 30 min, washed, and incubated for 30 min with Streptavidin Horseradish peroxidase conjugate followed by chemiluminescence detection. Compass evaluated SW 4.1.0.

### Colony formation assay

Cells were seeded (2 × 10^3^) in 6-well plates in medium containing 2.5% of CSS. Cells were treated daily, and the media were renewed every 2 days. After 10 days, cells were washed with PBS, fixed in acetone: methanol (1:1) for 3 min at room temperature, and stained with Crystal Violet for 20 min. A total of 10 pictures for each condition were detected using a digital camera, and the mean intensity of the colonies was measured by LICORbio ™.

### Scratch wound assay

Incucyte^®^ Imagelock 96-well Plate (Cat #BA-04856, Sartorius) was used to evaluate cell migration with the Scratch Wound technique. In 100µL in medium containing 2.5% CSS, ERα-positive BC cells (5 × 10^3^) were placed into each coated Incucyte^®^ Imagelock Plate well. Cells were left to settle on the bottom of the plate at room temperature for 15 min; then, the plate was incubated at 37 °C, 5% CO2 overnight. On the second day, the Incucyte^®^ Cell Migration Kit (Cat #BA-04858, Sartorium) was used simultaneously to create wounds in all wells. After wounding, cells were washed twice with dPBS, and the cells were exposed to treatment in 100µL in a medium containing 2.5% CSS overnight. Migration activity was quantified using the Incucyte S3 platform (Sartorius).

### Chemotaxis cell invasion assay

Incucyte^®^ Clearview 96-well Plate for Chemotaxis (Cat #4582, Sartorium) was used to evaluate in vitro cell invasion. The plate has been previously permeabilized with 150µL of dPBS and incubated for 20 min at 4° C according to the manufacturer’s instructions. ERα-positive BC cells (5 × 10^4^/mL) were mixed in a medium containing 2.5% CSS with Matrigel (1:20), and 20µL of matrix-cell suspension (5 × 10^3^) was dispensed into the insert. The plate was centrifuged for 3 minutes at 50 x g and incubated at room temperature for 60 min to allow the polymerization of the cell suspension. Afterward, 40µL of medium serum-free was added to the polymerized matrix-cell layer. On the bottom were 200 µL of medium 5% FBS containing the treatment. After 24 h, the invasion activity of live cells was quantified using the Incucyte S3 platform.

### Cell cycle analysis

To analyze cell cycle distribution, cells (1 × 10^5^) were cultured in regular medium in 6-well plates and shifted to medium without serum for 24 h. Next, cells were exposed to treatments, then pelleted, washed with PBS and fixed in PBS: methanol (1:1) overnight at − 20 °C before staining with a solution containing 50 µg/ml propidium iodide (PI) in 1×PBS, 20 U/ml RNAse-A and 0.1% Triton X-100. Cell phases were estimated as a percentage of a total of 10,000 events. Samples were then analyzed with FlowJo Software [37].

### Proliferation assay

Cells (2 × 10^3^) were seeded in 96-well plates in a regular growth medium, washed once they had attached, incubated in a medium containing 2.5% CSS, and then exposed to treatments. Cells were treated with 1µM niraparib and then exposed to treatments. Treatments were renewed every day for five days. Proliferation was quantified using the Incucyte S3 platform.

### Chromatin immunoprecipitation (ChIP)

After culturing in 10% CSS for 48 hours, cells were treated with 1µM fulvestrant and then exposed to treatments for 8 hours before to be harvested in ice-cold PBS. Cells were crosslinked in 1% formaldehyde in PBS. Crosslinking was quenched by adding glycine at a final concentration of 125 mM. Crosslinked cell pellets were snap-frozen and stored at − 80°C. For each ChIP experimental replicate, crosslinked cells (from 2 crosslinked aliquots) were lysed in lysis buffer with Pics using sonication (high, 30 seconds on/off, for 4 intervals of 10 minutes). 5% of lysate was reserved for input control and snap frozen to store at − 80°C. Lysates were diluted to 1 µg/uL protein based on Nanodrop A280 concentrations and divided into 1 mL aliquots. 5 ug of the appropriate antibodies (Epicypher ERα C-terminal for ERα ChIP and rabbit IgG for ERα negative control) were added to the appropriate lysate aliquots and rotated at 4°C overnight. Protein-chromatin was isolated and eluted using protein G beads (Cytiva Streptavidin Mag Sepharose™ Magnetic Bead, ThermoFisher Scientific). The samples were diluted in 1 ml of dilution buffer with pics and incubated overnight with Sp1 antibody. Again, Protein-chromatin was isolated and eluted using protein G beads. Eluted ChIP samples were incubated with RNAse A and Proteinase K to reverse the crosslinked protein-chromatin. Input samples and ChIP DNA were purified using a Qiagen QIAquick PCR Purification Kit, and purified DNA samples were eluted in 30 uL nuclease-free water. Input and ChIP purified DNA were quantified using IDT primers specific for probable regions of PARP-1 of shared chromatin binding by ERα and Sp1. Primer used are: R18S FWD (5’-GAGTGTTCAAAGCAGGTCCAA-3’); R18S REV (5’-CCTCTAGCGGTGCAATACAAA-3’); Sp1 FWD (5’-TGGGGCATTCCCATTAAGCA-3’); Sp1 REV (5’-TCTTCACAGTGCAAGGTCCC-3’). Quantabio PerfeCta^®^ SYBR^®^ Green (FastMix Reaction Mix with ROX™, ThermoFisher Scientific) was used for qPCR reactions using a Roche Step-One Real-Time PCR machine. qPCR Ct results were averaged and normalized to the endogenous control R18S.

### Chip-seq preparation and ChIP-seq pipeline

ChIP-DNA was quantitated by qubit, libraries were generated using the Illumina ChIP-SEQ kit and qc-ed by Bio-analyzer. Sequencing was on the Illumina NovaSEQ-X by the University of Chicago Functional Genomics core. Samples were analyzed over 50 M clusters / sample, which was variable due to the available library content. ChIP-seq data were uploaded to the Galaxy platform and analyzed using the public server at galaxy.org [38]. The ChIP-seq data quality was assessed using the FastQC tool. Sequencing files were mapped to the GRCh38 - hg38 human reference genome downloaded by UCSC Genome Browser Gateway and processed using Bowtie2. The data were converted to SAM and BAM formats and subsequently processed to evaluate the correlation among sample replicates using the multiBamSummary and plotCorrelation settings. The success of ChIP experiments was determined by analyzing the distribution of SES Fingerprint Graphs. The aligned reads were subsequently filtered for quality and uniquely mappable reads using Samtools. Library complexity was measured using BEDTools [39, 40]. Relaxed peaks were called using MACS with a *P* = 1 × 10 − 2 for each replicate [41]. To identify the ERα and FoxA1 binding sites affected by PARP-1, we used BEDTools to find the closest peaks from PARP-1–affected target genes compared with siPARP-1.

### Data collection

Bioinformatics analyses were performed on R Studio (version 2024.4.2.764) using the publicly available dataset Molecular Taxonomy of Breast Cancer International Consortium (METABRIC) [42]. The clinical information and the microarray mRNA expression data (Log2 transformed intensity values) of the METABRIC cohort were retrieved from cBioPortal for Cancer Genomics (http://www.cbioportal.org/) on March 4th, 2024. The analyses were carried out on estrogen receptor ERα-positive BC patients (n. 1506).

### Correlation and enrichment analyses

The Pearson correlation coefficients (r-values) between the expression levels of PARP-1 and the other genes of the METABRIC dataset were assessed in R Studio using the cor.test() function and setting the method as “Pearson”. The statistical significance of the correlation coefficients was calculated by the t-test, *p* < 0.0001 was considered as a cut-off criterion. The first 500 positively correlated genes were selected for the next evaluations. Aiming to cluster these genes in pathways, the ReactomePA package [33] was employed, setting the following parameters: organism = “human”, p-value cut-off = 0.05. Moreover, gene ontology (GO) analysis of biological process (BP) was performed by uploading our gene list on the Database for Annotation, Visualization and Integrated Discovery (DAVID) functional annotation analysis website [43]. The following parameters were chosen in the upload options: “official gene symbol” as identifier, “gene list” as list type, and “Homo sapiens” as species limit for the background.

### Survival analyses

The survival analyses on BC patients were assessed using PARP-1 gene expression data of the METABRIC cohort. Samples were filtered for missing values, ER-status and the vital status. As patients classified as “died of other causes” were excluded, we referred to “breast cancer specific survival” (BCS). The analyses were carried out on ERα-positive BC patients using the BCS and relapse-free survival (RFS) information. A log-rank test was used to determine differences between the survival curves. The survival analysis was performed using the survival [44] and the survminer [45] R packages. *p* < 0.05 was considered statistically significant.

### Statistical analysis

Biological triplicates were completed for each experiment (*n* = 3). Unless otherwise noted, data were analyzed by ordinary two-way ANOVA (α = 0.05) with Tukey’s multiple comparisons tests to compare between treatments within each cell line and between cell lines for each treatment. For all analyses: *p < 0.05, **p < 0.005, ***p < 0.0005, or ****p < 0.0001. For graphs, data points indicate the mean value of 3 experimental replicates, and error bars represent standard error (SE).

## Results

### PARP-1 is up-regulated by E_2_ in ERα-positive BC cells

**Fig. 1:**
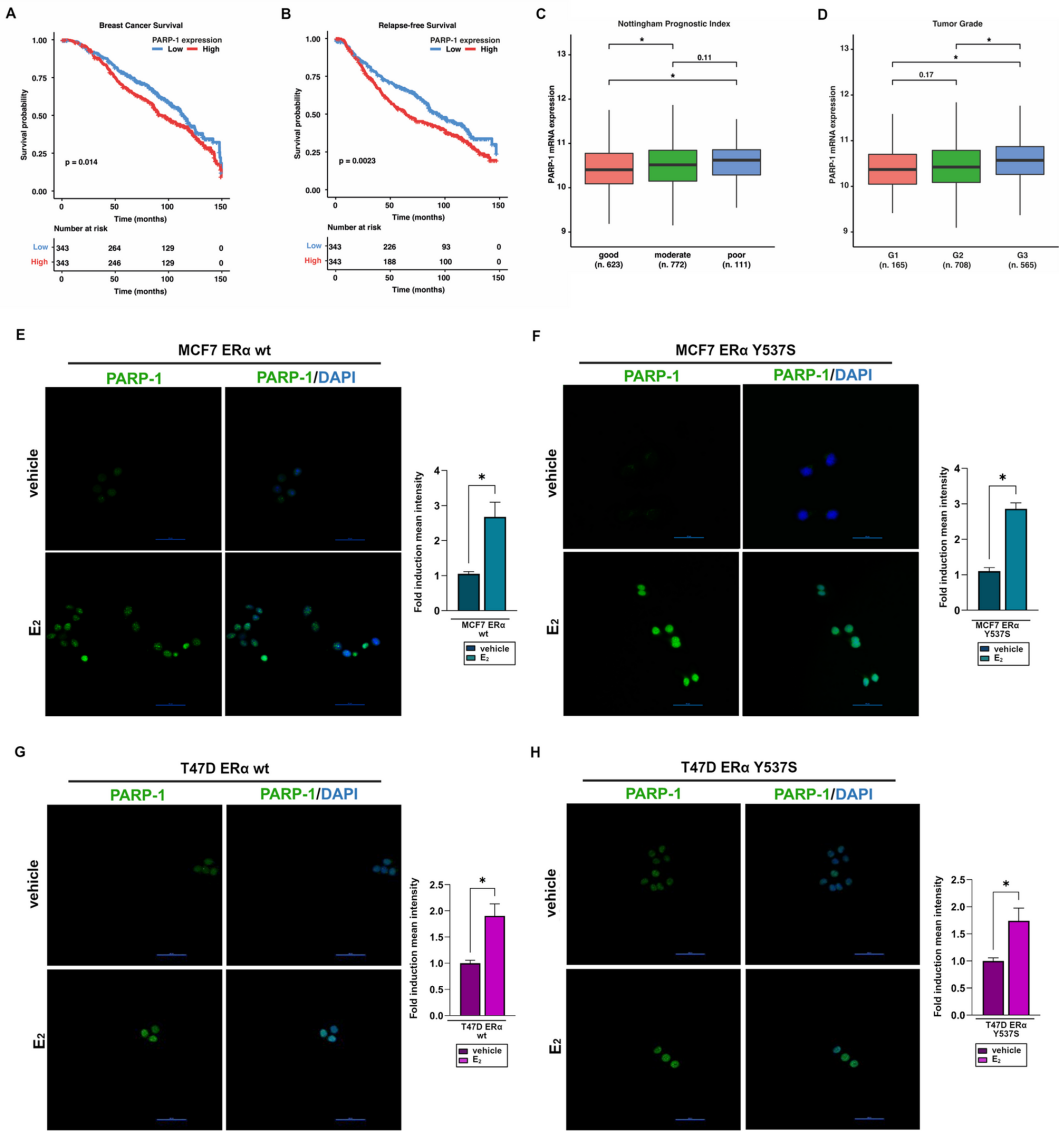
PARP-1 expression correlates with poor outcomes in ERα-positive breast cancer (BC) patients and is up-regulated by estrogen in ERα-positive BC cells. Kaplan-Meier curves depicting the correlation of PARP-1 levels with breast cancer survival (**A**) and relapse-free survival (**B**) in ERα-positive BC patients in the METABRIC dataset. BC patients who died from other diseases were not included in the analyses. Box plots showing the expression levels PARP-1 in ERα-positive BC patients in the METABRIC cohort, stratified by the Nottingham Prognostic Index (**C**) and the tumor grade (**D**). The number of patients is indicated in the panels. (*) *p*-value < 0.001. PARP-1 expression (green signal) evaluated by immunofluorescence experiments in ERα wild type (wt) MCF7 and T47D cells (**E**, **G**) or ERα Y537S mutated cells (**F**, **H**) treated with vehicle or 10 nM 17β-estradiol (E_2_) for 8 h. Nuclei were stained with 4′,6-diamidino-2-phenylindole dihydrochloride (DAPI, blue signal). The images represent 10 random fields from three independent experiments. Scale bar: 50 μm. The side panels represent the fold induction of mean fluorescent intensity of PARP-1 expression in E_2_ respect to vehicle-treated cells, calculated on at least 10 random fields of each sample. Data represent the average of three biological replicates, error bars indicate SEM. (*) *p* < 0.05

In ET-resistant BC patients, the ERα Y537S mutation appears in approximately 30% of circulating tumor cells and in more than 20% of metastatic tumors [46]. The identification of new pharmacological targets in these patients is a major focus of ongoing research. Recent studies have shown a role for the nuclear enzyme PARP-1 in BC cells and in the clinical outcomes of BC patients [20]. PARP-1 plays a pivotal role in numerous cellular signaling pathways, including DNA replication, transcription, chromatin remodeling, telomere integrity, cell survival and death [47–49]. Based on previous evidence showing a critical role for PARP-1 in BC progression through the regulation of oxidative DNA damage and the resistance to pharmacological treatments [50, 51], we aimed to explore the potential clinical significance of PARP-1 in BC patients using the METABRIC database. High levels of PARP-1 were significantly associated with worse BC survival (BCS) (Fig. 1A) and relapse-free survival (RFS) (Fig. 1B) in ERα-positive BC patients. In addition, elevated levels of PARP-1 expression represent an unfavorable prognostic indicator, as reflected by the correlation with a high Nottingham Prognostic Index (Fig. 1C) and tumor grade (Fig. 1D).

**Fig. 2:**
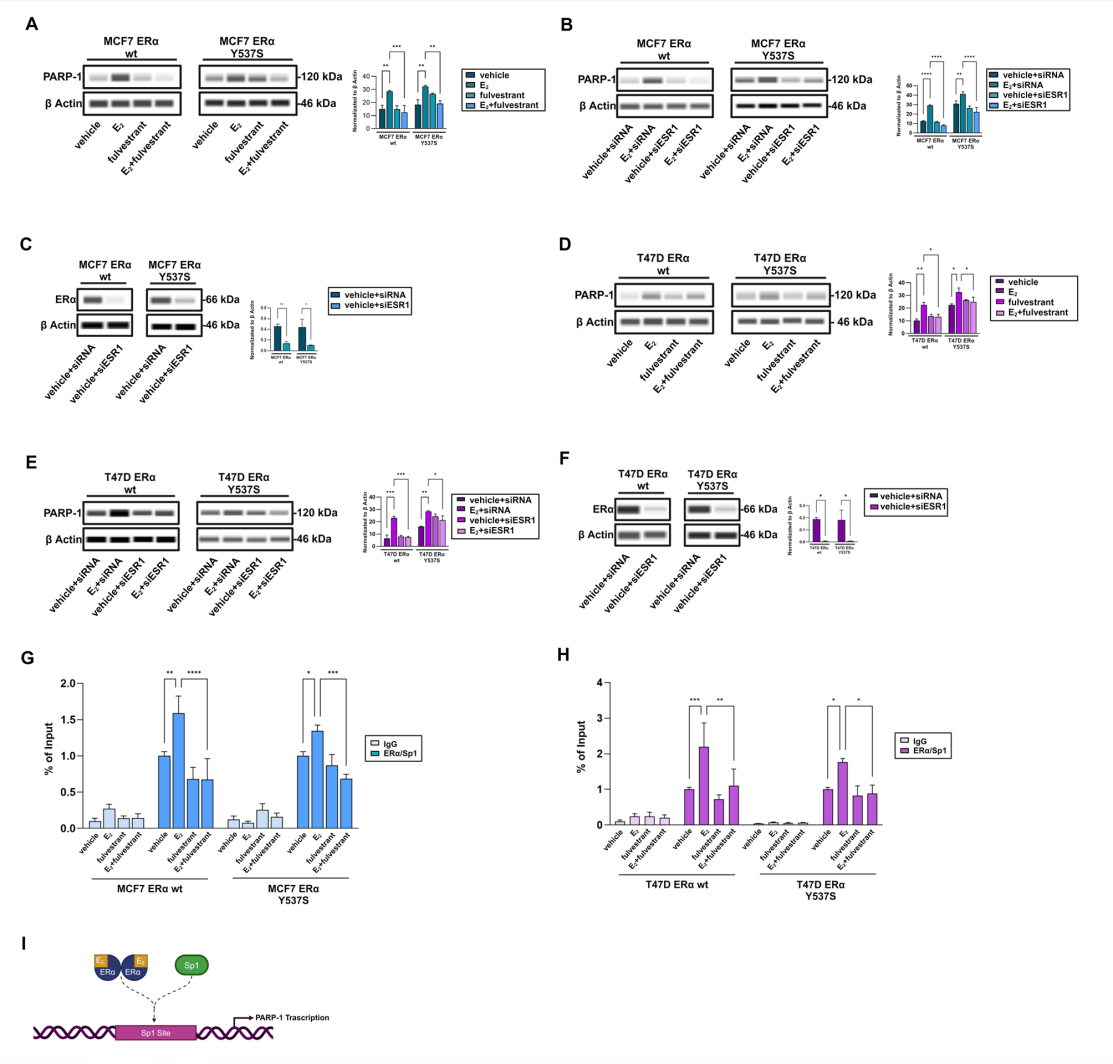
ERα is involved in estrogen-induced expression of PARP-1. (**A**, **D**) PARP-1 expression evaluated by immunoblotting assays in ERα wild type (wt) or Y537S mutated MCF7 or T47D cells treated for 8 h with vehicle or 10 nM 17β-estradiol (E_2_) alone or in combination with 1µM fulvestrant. Immunoblots of PARP-1 (**B**, **E**) and ERα (**C**, **F**) levels in ERα wt or Y537S mutated MCF7 or T47D cells transfected with siRNA or siESR1 (encoding for ERα) and treated with vehicle or 10 nM E_2_ for 8 h, as indicated. Right panels show densitometric analysis of the blots normalized to β-Actin, which was used as a loading control. Recruitment of ERα and Sp1 to PARP-1 promoter by ChIP assay in ERα wt or Y537S mutated MCF7 (**G**) and T47D (**H**) cells. In control samples, nonspecific IgGs were used instead of the primary antibody. The amplified sequences were evaluated by real-time PCR. Data represent the average of three biological replicates with error bars indicating SEM. (**I**) Diagram depicting PARP-1 chromatin site assessed for ERα and Sp1 binding using chromatin immunoprecipitation (ChIP) created with Biorender.com. (*) *p* < 0.05; (**) *p* < 0.005; (***) *p* < 0.0005; (****) *p* < 0.0001

Based on these observations, we focused on the mechanisms involved in the regulation of PARP-1 expression and activity in ERα-positive BC cells. First, real-time PCR and immunofluorescence analyses showed that 17β-estradiol (E_2_) significantly increases the mRNA and protein levels of PARP-1 in MCF7 and T47D cells expressing ERα wild type (Additional File 1 A-B; Fig. 1E, G) or Y537S mutation (Additional File 1 A-B; Fig. 1F, H). Next, we investigated the potential involvement of ERα in the modulation of PARP-1 in MCF7 and T47D BC cells expressing ERα wild type or Y537S mutation. Of note, we observed that the up-regulation of PARP-1 protein levels induced by E_2_ is prevented by the ERα antagonist fulvestrant (Fig. 2A, D) and by silencing ERα expression with a specific siRNA (Fig. 2B-C, E-F). These findings indicate that ERα signaling regulates the expression of PARP-1 in BC cells expressing either ERα wild type or Y537S mutation. It is well known that activated ERα binds to the Estrogen Response Elements (EREs) associated with target genes [52–54]. However, transcriptional modulation of ERα target genes can also occur through the involvement of other transcription factors like activator protein 1 (AP1), specific protein 1 (Sp1) and nuclear factor κB (NFκB) [55–58]. Previous studies have shown that ERα can interact with the C-terminal DNA binding domain of Sp1 [59] and the binding of ERα/Sp1 complex to Sp1 sites located within the promoters of certain genes induces transcriptional changes in BC cells upon estrogen treatment [60]. Considering that many sites are responsive to Sp1 in the PARP-1 promoter sequence, we investigated the role of Sp1 in PARP-1 modulation by E_2_. Of note, re-ChIP assays showed that E_2_ promotes the recruitment of both Sp1 and ERα to the Sp1 site located within the PARP-1 promoter region of MCF7 (Fig. 2G) and T47D (Fig. 2H) cells expressing ERα wild type or Y537S mutation. Further corroborating the involvement of ligand-induced ERα activation in this response, the recruitment of Sp1 and ERα to the PARP-1 promoter region was prevented using fulvestrant in the aforementioned BC cells (Fig. 2G-I).

### PARP-1 is involved in the transcriptional activity of ERα

**Fig. 3:**
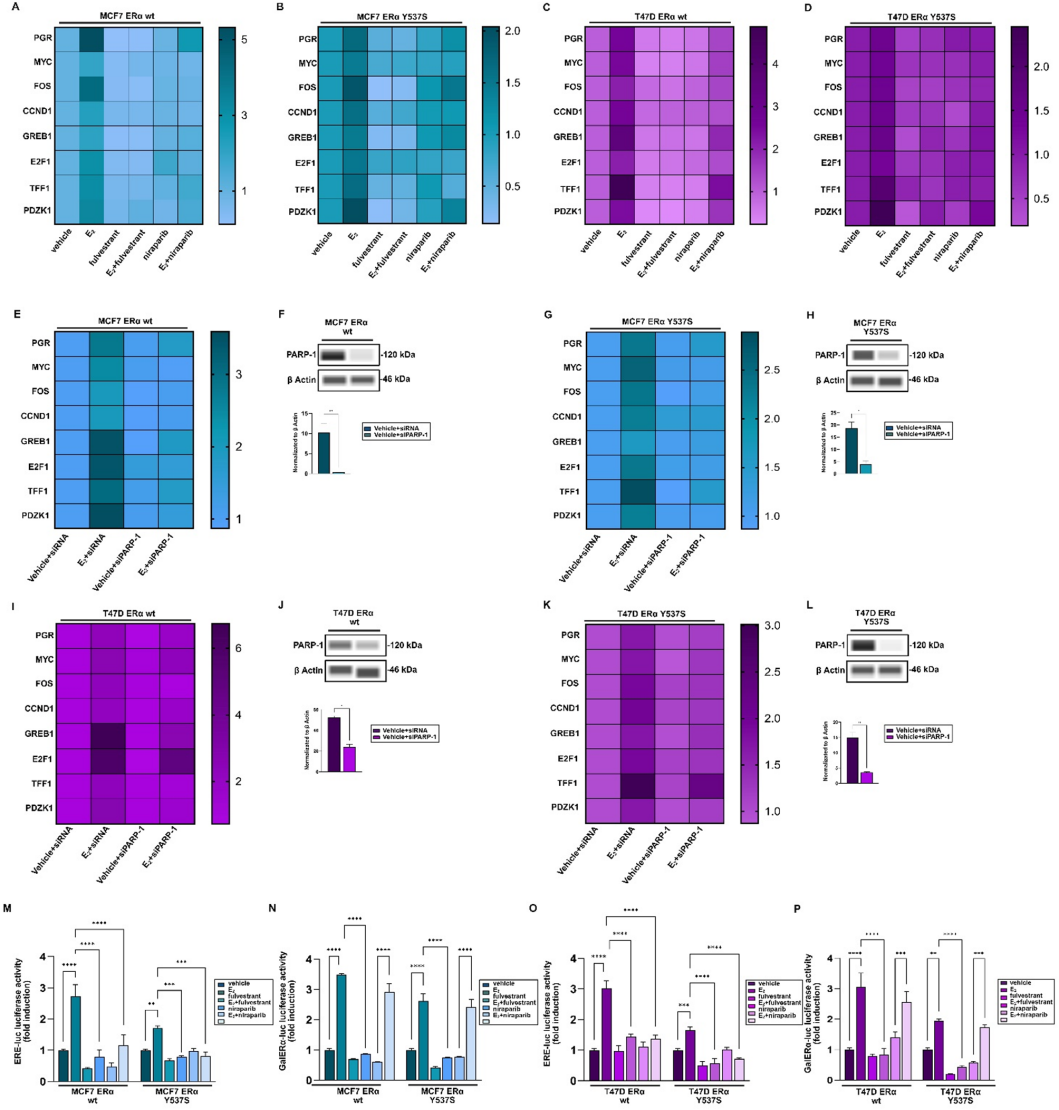
PARP-1 regulates the transcriptional activity of ERα in BC cells. Heatmaps of mRNA expression of main ERα target genes evaluated by real-time PCR in ERα wild type (wt) or Y537S mutated MCF7 (**A-B**) and T47D (**C-D**) cells treated for 24 h with vehicle or 10 nM E_2_ alone or in combination with 1µM fulvestrant or niraparib, as indicated. mRNA expression of major ERα target genes in ERα wt (**E**, **I**) and Y537S mutated (**G**, **K**) MCF7 and T47D cells. Cells were transiently transfected for 36 h with negative control (siRNA) or siPARP-1 and treated with vehicle or 10 nM 17β-estradiol (E_2_), as indicated. Values are normalized to human beta-2-microglobulin (B2M) endogenous control expression and shown as fold changes of mRNA expression. Efficacy of PARP-1 silencing in ERα wt (**F**, **J**) and Y537S mutated (**H**, **L**) MCF7 and T47D cells. Side panels show a densitometric analysis of the blots normalized to β Actin, which was used as a loading control. (**M-P**) ERα wt and Y537S mutated MCF7 and T47D cells were transfected with an ER luciferase reporter gene (ERE-luc) along with the internal transfection control Renilla Luciferase (**M**, **O**) or with Gal4 reporter gene GK1, the Gal4 fusion protein encoding the Ligand Binding Domain (LBD) of ERα (GalERα) and the internal transfection control Renilla Luciferase (**N**, **P**), and next were treated for 12 h with vehicle or 10 nM E_2_ in the presence or absence of 1µM fulvestrant or niraparib, as indicated. The normalized luciferase activity values of cells treated with vehicle (-) were set as 1-fold induction, upon which the activity induced by treatments was calculated. Data represent the average of three biological replicates with error bars indicating SEM. (**) *p* < 0.005; (***) *p* < 0.0005; (****) *p* < 0.0001

Based on previous evidence showing a role for PARP-1 in modulating both ERα transcriptional activity and ET in ERα-positive BC cells [20, 61], we evaluated the expression of ERα target genes upon E_2_ exposure either in the presence of fulvestrant or the PARP-1 inhibitor niraparib in BC cells expressing ERα wild type or Y537S mutation. Real-time PCR studies revealed that both inhibitors reduced the expression of diverse ERα target genes prompted by E_2_, including PGR, MYC, FOS, CCND1, GREB1, E2F1, TFF1 and PDZK1, in both ERα wild type and Y537S mutated MCF7 (Fig. 3A-B) and T47D (Fig. 3C-D) cells. Further corroborating the involvement of PARP-1 activity in these effects, we observed similar results by silencing PARP-1 expression in MCF7 (Fig. 3E-H) and T47D (Fig. 3I-L) ERα wild type and Y537S mutated BC cells. We also investigated the potential involvement of PARP-2, an additional member of the PARP family, in the regulation of ERα transcriptional activity. As PARP-2 shares the catalytic domain with PARP-1, both enzymes play an important role in DNA repair mechanisms by regulating similar functions [62, 63]. As expected, PAPR-2 silencing reduced the expression of major ERα target genes induced by E_2_ in both ERα wild type and Y537S mutated BC cells (Additional File 2).

To further demonstrate the involvement of PARP-1 in regulating the transcriptional activity of ERα, we performed luciferase reporter assays in MCF7 and T47D cells transiently transfected with an ERα reporter gene (ERE-luc) or a chimeric protein consisting of the DNA binding domain (DBD) of the yeast transcription factor Gal4 and the LBD of ERα (GalERα-luc) (Additional File 3) [64]. Of note, we found that either fulvestrant or niraparib inhibited the E_2_-stimulated transactivation of the ERE-luc construct in both ERα wild type and Y537S mutated cells (Fig. 3M, O). Notably, niraparib failed to prevent the transactivation of the GalERα-luc plasmid triggered by E_2_ (Fig. 3N, P), suggesting the involvement of PARP-1 in the transcriptional activity of ERα, but not via interaction with its cognate ligand.

**Fig. 4:**
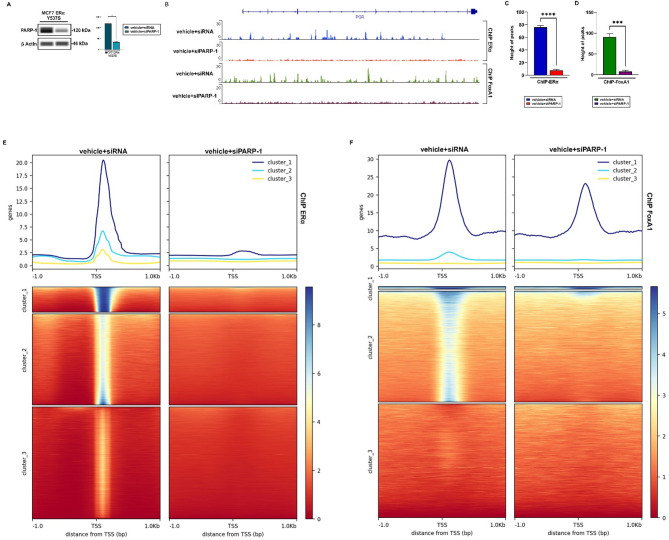
PARP-1 influences the DNA binding between ERα and FoxA1. (**A**) Efficacy of PARP-1 silencing in ERα Y537S mutated MCF7 cells. Side panel shows a densitometric analysis of the blots normalized to β-Actin, which was used as a loading control. (**B**) ChIP-seq analysis showing ERα and FoxA1 binding in MCF7 ERα Y537S of the PGR gene. The peaks of ChIP-seq were analyzed via IGV.org platform. Analysis of ERα (**C**) or FoxA1 (**D**) DNA binding peaks in MCF7 ERα Y537S cells transfected with siRNA or siPARP-1. Data represent the average of 3 biological replicates with error bars indicating SEM. (*) *p* < 0.05; (***) *p* < 0.0005; (****) *p* < 0.0001. Heat maps of global evaluation enrichment around the Transcription Start Site (TSS) in ChIP of ERα (**E**) or FoxA1 (**F**) in MCF7 ERα Y537S cells transfected for 30 h with siRNA or siPARP-1. Sequencing data was analyzed via the galaxy.org platform

Considering recent evidence showing a role for the ERα co-activator FoxA1 in the PARP-1-mediated regulation of estrogen target genes in BC cells [20, 65–68], we evaluated whether these molecular events may also occur in ERα Y537S mutated BC cells. Thus, we performed ChIP-Sequencing (ChIP-Seq) studies by immunoprecipitating ERα or FoxA1 in ERα Y537S mutated MCF7 cells silenced or not for PARP-1 expression (Fig. 4A). We observed a significant increase of either ERα or FoxA1 signal at the promoter of the ERα target gene namely PGR (Fig. 4B). Of note, PARP-1 silencing abrogated these responses, suggesting that PARP-1 is involved in the binding of ERα or FoxA1 to the promoter regions of ERα target genes. Moreover, the interaction peaks between both ERα and FoxA1 and the DNA in ERα Y537S mutated MCF7 cells were reduced when PARP-1 expression was silenced (Fig. 4C-D). Data obtained from ChIP-seq assays were also used to compare the interaction between ERα or FoxA1 and the DNA in MCF7 ERα Y537S mutated BC cells silenced for PARP-1 expression. The heatmaps were created by examining the Transcription Start Site (TSS) from − 1000/+1000 region surrounding the TSS. Genes were divided into clusters based on the presence or absence of a promoter-associated CpG island. The results obtained demonstrate that ERα and FoxA1 bind to numerous overlapping sites on gene promoters in ERα Y537S mutated MCF7 cells, however a reduced interaction of both proteins with the DNA occurs silencing PARP-1 expression (Fig. 4E-F).

### Biological role of PARP-1 in ERα wild type and Y537S mutated BC cells

To better appreciate the biological significance of PARP-1 in breast malignancy [69], we explored the gene expression landscape associated with high PARP-1 levels in ERα-positive BC patients of the METABRIC dataset.

**Fig. 5:**
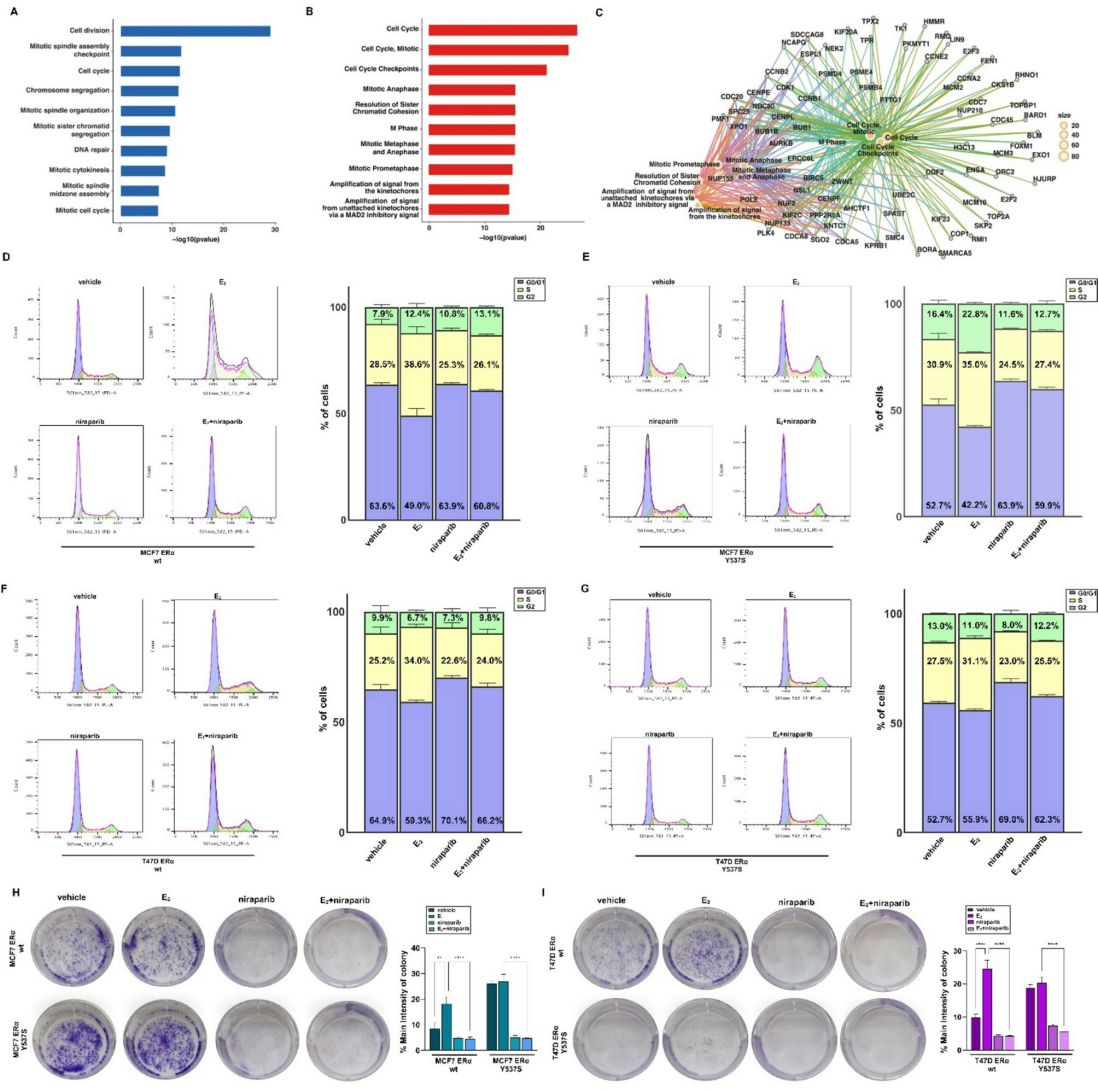
PARP-1 is implicated in the proliferative behavior of ERα wt and Y537S mutated BC cells. (**A**) Bar plot displaying the ten most significant PARP-1-related biological processes resulting from gene ontology analysis, which included the top 500 genes positively correlated to PARP-1 (by Pearson correlation analysis) in ERα-positive BC patients of the METABRIC dataset. The number of genes included in each term is displayed within the bars. p-value < 0.05 was set as a significant threshold. (**B**) Bar plot displaying the ten most significant PARP-1‐related pathways from Reactome pathway analysis using the top 500 genes positively correlated to PARP-1 (by Pearson correlation analysis) in ERα-positive BC patients of the METABRIC dataset. The color bar gradient indicates the range of significance (BH p‐adjusted values) of each pathway. p-adjusted < 0.05 was set as a significant threshold. (**C**) Interrelation network representing the genes correlated to PARP-1 included in the enriched pathways previously identified through Reactome analysis. (**D-G**) Cell cycle analysis performed by flow cytometry assays in ERα wild type (wt) and Y537S mutated MCF7 (**D-E**) and T47D cells (**F-G**) cells treated with vehicle or 10 nM of E_2_ for 12 h alone or in combination with 1µM niraparib. Side panels show the percentage of cells in G0/G1, S and G2/M phases of the cell cycle. Colony formation in MCF7 (**H**) and T47D (**I**) cells expressing ERα wt or Y537S mutated exposed to vehicle or 10 nM E_2_ alone or in combination with 1µM niraparib. The plates were stained with Crystal Violet. After 10 days of incubation cell colonies were stained and pictures were captured by a digital camera. Colonies were counted using the program LICORbio. Data represent the average of three biological replicates with error bars indicating SEM. (**) *p* < 0.005; (****) *p* < 0.0001

We performed gene ontology (GO) and pathway enrichment analyses using the 500 genes that most correlated with PARP-1 expression. These genes were found to be associated with both GO terms (Fig. 5A) and transduction pathways (Fig. 5B-C) involved in the proliferation and cell cycle regulation of BC cells, suggesting that high levels of PARP-1 might facilitate the acquisition of aggressive features in ERα-positive BCs. Consistent with the above evidence, flow cytometric analyses showed that the E_2_-induced S-phase entry is lost in the presence of niraparib in ERα wild type MCF7 (Fig. 5D) and T47D (Fig. 5F) cells. Furthermore, niraparib lowered the percentage of vehicle-treated ERα Y537S mutated MCF7 (Fig. 5E) and T47D (Fig. 5G) cells in the S phase of the cell cycle. Similar responses were observed in proliferative (Additional File 4) and clonogenic (Fig. 5H-I) assays, suggesting that PARP-1 is involved in the growth and colony-forming abilities of both ERα wild type and Y537S mutated BC cells. In addition, we explored other potential biological effects mediated by PARP-1, such as migration and invasion. In accordance with previous evidence showing that many ERα-positive BC cells exhibit a low migratory potential [70], MCF7 expressing ERα wild type did not show motile properties (Additional File 5). In contrast, niraparib effectively suppressed the migratory (Additional File 5A) and invasive (Additional File 5B) potential of MCF7 ERα Y537S cells.

### Targeting both ERα and PARP-1 impairs BC growth

**Fig. 6:**
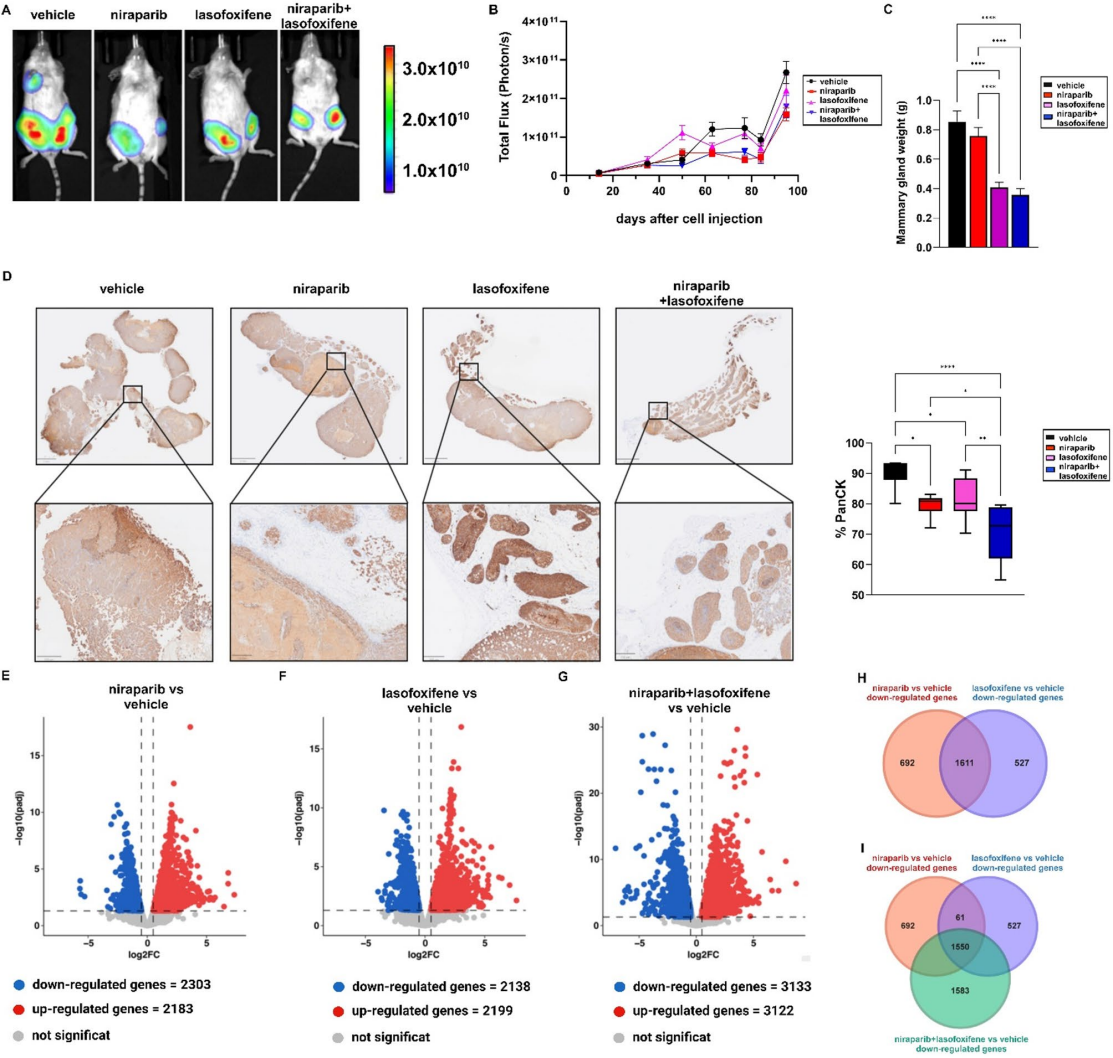
Effects of PARP-1 and ERα inhibition on the progression of BC tumors expressing ERα Y537S mutation. (**A**) Representative in vivo luminescence images of mice bearing tumors expressing ERα Y537S mutated MCF7 cells at day 90 after treatment initiation. (**B**) Total photon flux of luminescence signals measured by in vivo imaging for tumors expressing ERα Y537S mutated MCF7 cells (*n* = 8). (**C**) The average weight of mammary glands at the time of sacrifice (*n* = 8 glands). (**D**) Representative IHC sections of PanCK staining for each treatment mice group; right box plot of PanCK % in the mammary gland determined with QuPath for Windows. Data represent the average of biological replicates with error bars indicating SEM. (*) *p* < 0.05; (**) *p* < 0.005; (****) *p* < 0.0001. (**E**) Volcano plot illustrating the differentially expressed genes (DEGs) in niraparib respect to vehicle-treated ERα Y537S MCF7-derived tumors. (**F**) Volcano plot showing the DEGs in lasofoxifene respect to vehicle-treated ERα Y537S MCF7-derived tumors. (**G**) Volcano plot illustrating the DEGs in niraparib + lasofoxifene respect to vehicle-treated ERα Y537S MCF7-derived tumors. Significantly down-regulated genes (log2FC ≤ -0.5 and padj < 0.05) are shown in blue, significantly up-regulated genes (log2FC ≥ 0.5 and padj < 0.05) are shown in red, non-significant genes are shown in grey (padj ≥ 0.05). (**H**) Venn diagram showing the intersection of the genes down-regulated in niraparib and lasofoxifene-treated ERα Y537S MCF7-derived tumors respect to the vehicle-treated counterpart. (**I**) Venn diagram showing the intersection of the genes commonly down-regulated by niraparib, lasofoxifene and niraparib + lasofoxifene respect to vehicle in ERα Y537S MCF7-derived tumors

Recent studies have shown promising results using the SERM lasofoxifene in ERα Y537S mutated cells compared to other drugs like fulvestrant and the CDK4/6 inhibitor palbociclib, which are widely used in clinical settings [71, 72]. Therefore, we evaluated the efficacy of lasofoxifene in combination with niraparib in mouse xenograft models derived from ERα Y537S mutated MCF7 cells. Notably, real-time luminescence images (Fig. 6A) and the total photon flux quantification of the signal (Fig. 6B) indicated that both lasofoxifene and niraparib (5 and 10 mg/kg, respectively) reduced the primary tumor mass compared to the vehicle-treated xenografts. As the mammary gland weights were not significantly altered in mice treated with niraparib (Fig. 6C), we performed Pan CK (Fig. 6D) and H&E (Additional File 6) staining and observed significant areas of necrotic gland tissues.

To provide further evidence for a key role of PARP-1 in mediating the proliferative behavior of ERα-positive tumors, we expanded our in vitro analyses by evaluating an additional PAPR-1 inhibitor in the presence or absence of lasofoxifene. Specifically, growth assays were performed in ERα wild type and Y537S mutated BC cells treated with olaparib, either alone or in combination with lasofoxifene. The results indicate that olaparib, similar to niraparib, elicits anti-proliferative effects in BC cells expressing ERα wild type and the Y537S mutation (Additional File 7). Treatment with niraparib and/or lasofoxifene significantly reduced the expression of the proliferative marker Ki67 in tumor xenografts derived from ERα Y537S mutated MCF7 cells (Additional File 8 A). In agreement with these data, bioinformatics analyses showed a correlation between PARP-1 and Ki67 expression levels in ERα-positive BC patients of the METABRIC dataset (Additional File 8B). To better understand the global gene expression networks regulated by niraparib and lasofoxifene either alone or in combination, we performed RNA-seq analyses in tumors derived from mice injected with ERα Y537S mutated MCF7 cells. The differentially expressed genes (DEGs) were identified based on the following criteria: log2FC ≥ 0.5 or log2FC ≤ -0.5, and adjusted p-value < 0.05 (Fig. 6E-G). To assess the biological significance of the DEGs in ERα Y537S mutated cells, we performed pathway enrichment analysis, focusing on the down-regulated genes under three different conditions: niraparib versus vehicle (Additional Table 1), lasofoxifene versus vehicle (Additional Table 2) and niraparib plus lasofoxifene versus vehicle (Additional Table 3). Remarkably, niraparib induced a significant down-regulation of ESR1 as well as genes belonging to the estrogen-signaling pathway. Finally, to assess the transcriptional changes shared by niraparib and lasofoxifene, we compared the genes downregulated by both inhibitors. The Venn diagrams in Fig. 6 (panel H) show the number of the common down-regulated genes in samples treated with niraparib and lasofoxifene (Fig. 6H, I). Overall, these findings suggest that PARP-1 is involved in the transcriptional regulation of ERα target genes toward proliferative events in BC cells expressing Y537S mutation.

## Discussion

BC represents the most diagnosed cancer globally and its incidence is increased over the past few decades [2, 73]. Targeting ERα by ET is a primary therapeutic option in ERα-positive BC patients [74–76], however resistance to ET often develops, indicating that further efforts are needed to improve clinical outcomes [77]. Previous studies have demonstrated the role of acquired ESR1 mutations in ET resistance [16, 78–80]. Of note, these mutations have been identified in metastatic ERα-positive BC patients with a frequency ranging from 10 to 40% [13, 81]. Importantly, many drugs used to treat ERα-positive BC have significantly reduced therapeutic efficacy in the presence of the ERα Y537S mutation [17, 18]. The Y537S mutation is located at the beginning of H12 in the LBD, stabilizing the active conformation of the LBD [18, 46], which promotes the constitutive activity of AF2 in the absence of E_2_ [18]. In agreement with this observation, previous findings have highlighted that the activating Y537S ESR1 mutation promotes resistance to first-line ET [68, 82]. A comprehensive understanding of the biological responses triggered by ERα Y537S mutation in BC would be useful toward the assessment of novel therapeutic strategies.

The nuclear enzyme PARP-1 has recently attracted significant interest due to its critical role in tumor progression involving repair of oxidative DNA damage [83–86]. PARP-1 reverses the effects of DNA single-strand breaks (SSBs) through the process of base excision repair (BER) [87]. The c-terminal catalytic domain of PARP-1 enzyme transfers ADP-ribose subunits from NAD + to protein acceptors, which are widely involved in cellular differentiation, gene transcription, inflammation and cell death processes [88–90]. In response to SSBs, PARP-1 binds the sites of DNA damage, recruiting additional repair proteins to the damaged DNA site, including ataxia-telangiectasia mutated (ATM) and meiotic recombination 11 homolog A (MRE11) [47, 91]. Thus, PARP inhibitors are a promising therapeutic strategy for diverse types of tumors. In this context, preclinical research has demonstrated that BC cells expressing BRCA1/2 mutations exhibit sensitivity to PARP-1 inhibition due to their dependence on PARP-1 activity for DNA repair [92, 93]. In addition, PARP-1 has been shown to be involved in estrogen-induced gene transcription in ERα-positive BC cells [20]. Based on these findings, we first determined that elevated levels of PARP-1 are associated with a less favorable prognosis in ERα-positive BC patients. Subsequently, we found that E_2_ increases the expression of PARP-1 in both ERα wild type and Y537S mutated BC cells. In agreement with the known capacity of ligand-activated ERα to bind to EREs either alone or together with co-factors such as FoxA1 [94, 95] as well as to interact with other transcription factors like Sp1 [96], re-ChIP experiments demonstrated that ERα and Sp1 stimulate the transcription of the PARP-1 gene by binding to a Sp1 site located within its promoter sequence. According to previous data indicating that PARP-1 may regulate gene transcription [97, 98], the inhibition of PARP-1 prevented the E_2_-induced up-regulation of diverse ERα target genes in both ERα wild type and Y537S mutated BC cells.

**Fig. 7:**
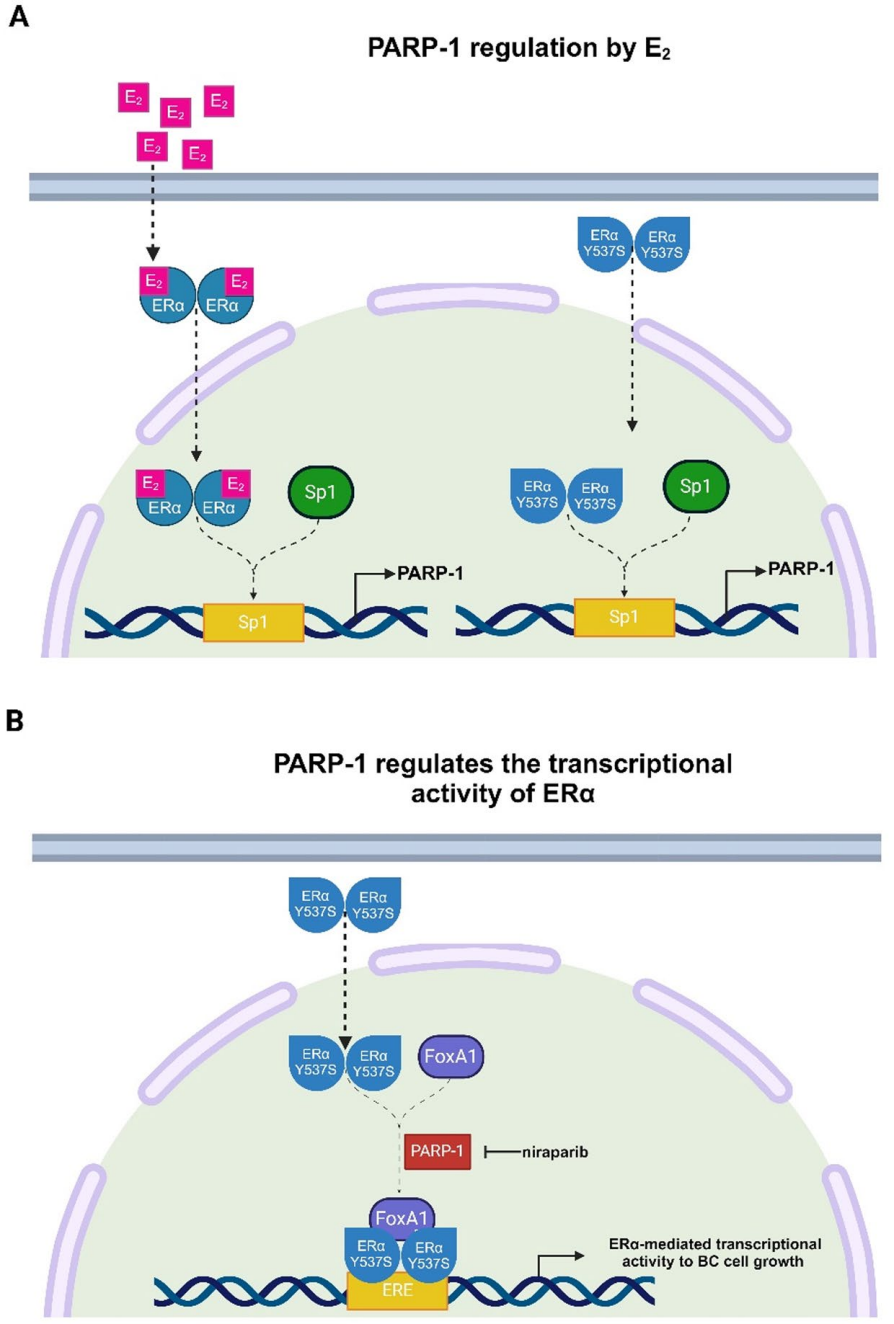
Proposed mechanisms involved in the regulation of PARP-1 by E_2_ and ERα transcriptional activity by PARP-1. (**A**) E_2_-induced (in ERα wt BC cells) or constitutive active (in ERα Y537S mutated BC cells) ERα dimerizes, translocate into the nucleus and interacts with the Sp1 protein on specific Sp1 DNA binding sites located within the promoter region of PARP-1, toward its transcription. (**B**) In ERα Y537S mutated BC cells, PARP-1 is involved in the regulation of the ERα and FoxA1-mediated gene expression machinery that is associated with proliferative effects

The forkhead protein FoxA1 is a pioneer factor involved in the interactions between ERα and chromatin sites [99]. Specifically, FoxA1 influences the global binding of ERα mediating responses to endocrine therapy as well as mechanisms of resistance in ERα-positive BC cells [100]. Some experimental evidence has revealed an important role exerted by FoxA1 on chromatin structure and the binding of transcriptional factors in ERα Y537S mutated BC cells [101]. In accordance with these and previous studies demonstrating that ADP-ribosylation may have an impact on the activity of ERα and its cofactor FoxA1 in BC cells [20], we observed that PARP-1 is implicated in the regulation of the gene expression machinery mediated by ERα and FoxA1 in ERα Y537S mutated BC cells (Fig. 7A-B). Our in vitro data also suggested that the inhibition of PARP-1 interferes with the proliferation of BC cells expressing either ERα or Y537S mutation. Additionally, we explored the role of PARP-1 inhibition in *in vivo models* expressing Y537S mutations. Importantly, niraparib prevented the growth of xenograft tumors derived from ERα Y537S mutated BC cells and exhibited an inhibitory action on whole gene transcription similar to the response observed using lasofoxifene.

## Conclusions

The current study provides novel insights into the molecular mechanisms through which PARP-1 may contribute to the growth of BC cells expressing ERα wild type or the constitutively active Y537S mutation. Our findings indicate that estrogen triggers the up-regulation of PARP-1, which in turn prompts a feed-forward loop enhancing the transcriptional activity of ERα promoting proliferative events. Therefore, PARP-1 should be included among the factors involved in the ERα-mediated processes that stimulate growth responses of ERα-positive BC cells, including those expressing the Y537S mutation. Finally, the use of PARP-1 inhibitors should be considered as a promising therapeutic strategy in BC patients whose tumors express ERα wild type or the Y537S mutation.

## Electronic supplementary material

Below is the link to the electronic supplementary material.

Supplementary Material 1

Supplementary Material 2

Supplementary Material 3

Supplementary Material 4

Supplementary Material 5

Supplementary Material 6

Supplementary Material 7

Supplementary Material 8

Supplementary Material 9

Supplementary Material 10

Supplementary Material 11

## Data Availability

All data that were generated or analyzed during our study have been included in this article. Materials, additional data and protocols described within the manuscript will be made available from the authors upon reasonable request. The raw data reported in this study are deposited in the GEO repository with accession number GSE277883.

## Abbreviations

AF-2: Activating function-2
AI: Aromatase inhibitor
AP-1: Activator protein 1
ATM: Ataxia-telangiectasia mutated
BC: Breast cancer
BCS: Breast cancer specific survival
BER: Base excision repair
CCND1: Cyclin D1
ChIP-seq: Chromatin immunoprecipitation-sequencing
DBD: DNA binding domain
DEGs: Differential expression genes
E2F1: E2F Transcription factor 1
ET: Endocrine therapy
ER: Estrogen receptor
EREs: Estrogen response elements
ERE-luc: ERαreporter gene
E_2_: 17β-Estradiol
FOS: Fos proto-oncogene
FoxA1: Forkhead box protein A1
GO: Gene ontology
Greb1: Growth regulating estrogen receptor binding 1
H12: Helix 12
H&E: Hematoxylin and eosin
LBD: Ligand binding domain
METABRIC: Molecular taxonomy of breast cancer international consortium
MRE11: Meiotic recombination 11 homolog A
MYC: Proto-oncogene, BHLH transcription factor
NFkB: Nuclear factor kappa B subunit 1
PARP-1: Poly (ADP-ribose) polymerase-1
PARP-2: Poly (ADP-ribose) polymerase-2
PDzK1: PDZ domain containing 1
PGR: Progesterone receptor
RFS: Relapse – free survival
RNA-seq: RNA-sequencing
SERM: Selective estrogen receptor
SERD: Selective estrogen receptor down-regulators
SP1: Sp1 transcriptional factor
SSBs: Single-strand breaks
TFF1: Trefoil factor 1
TSS: Transcription start site
