# Supplementary material for: PARP-1 as a novel target in endocrine-resistant breast cancer
Source: J Exp Clin Cancer Res. 2025 Jun 16;44:175. doi: 10.1186/s13046-025-03441-4 (PMC12168341; doi:10.1186/s13046-025-03441-4)
Supplement: Supplementary file 4 — Supplementary Material 4 [file 13046_2025_3441_MOESM4_ESM.docx]

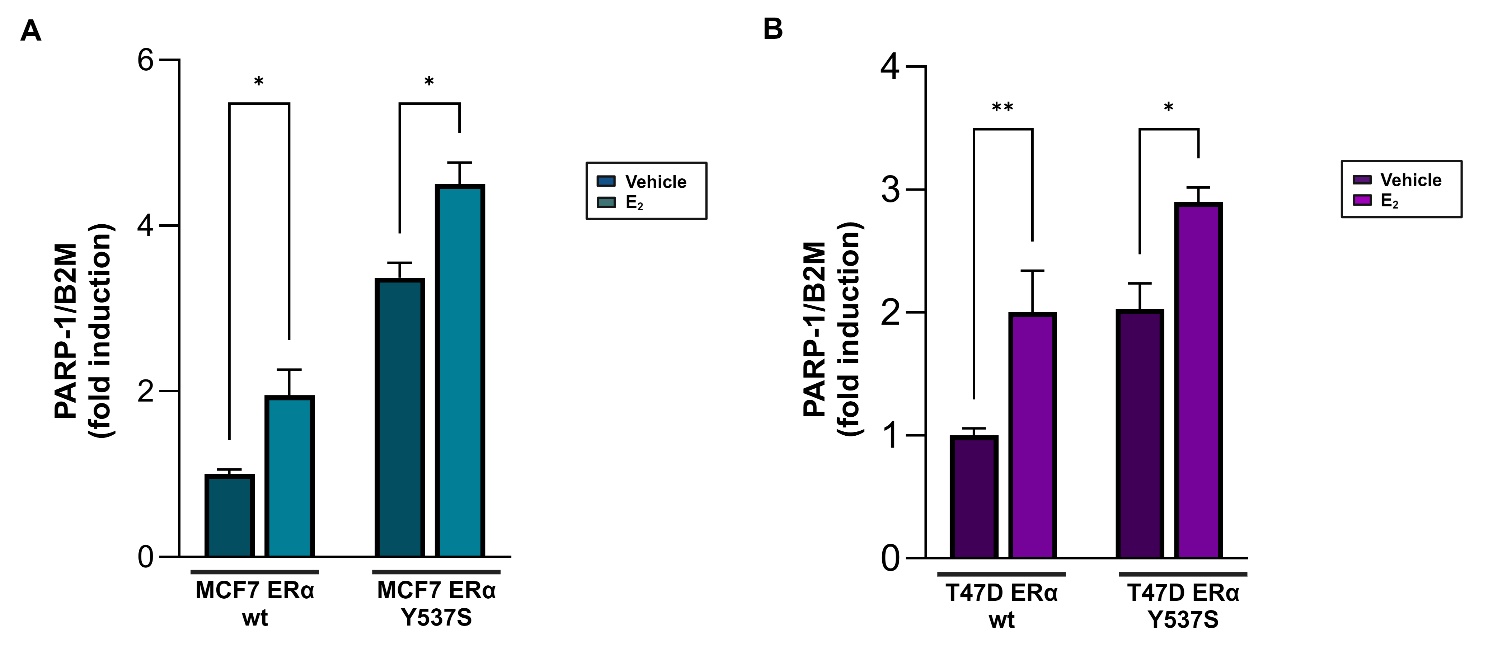


**Additional File 1. Estrogens increase PARP-1 mRNA levels in breast cancer cells.** mRNA expression of PARP-1 in ERα wild type (wt) and Y537S mutated MCF7 **(A)** and T47D **(B)** cells exposed to vehicle or 10nM of 17β-estradiol (E_2_) for 6 hours. Values are normalized to human beta-2-microglobulin (B2M) endogenous control expression and shown as fold changes of mRNA expression in E_2_ respect to vehicle-treated cells. Data represent the average of three biological replicates with error bars indicating SEM. (*) p < 0.05; (**) p < 0.005.
