# Supplementary material for: PARP-1 as a novel target in endocrine-resistant breast cancer
Source: J Exp Clin Cancer Res. 2025 Jun 16;44:175. doi: 10.1186/s13046-025-03441-4 (PMC12168341; doi:10.1186/s13046-025-03441-4)
Supplement: Supplementary file 5 — Supplementary Material 5 [file 13046_2025_3441_MOESM5_ESM.docx]

**
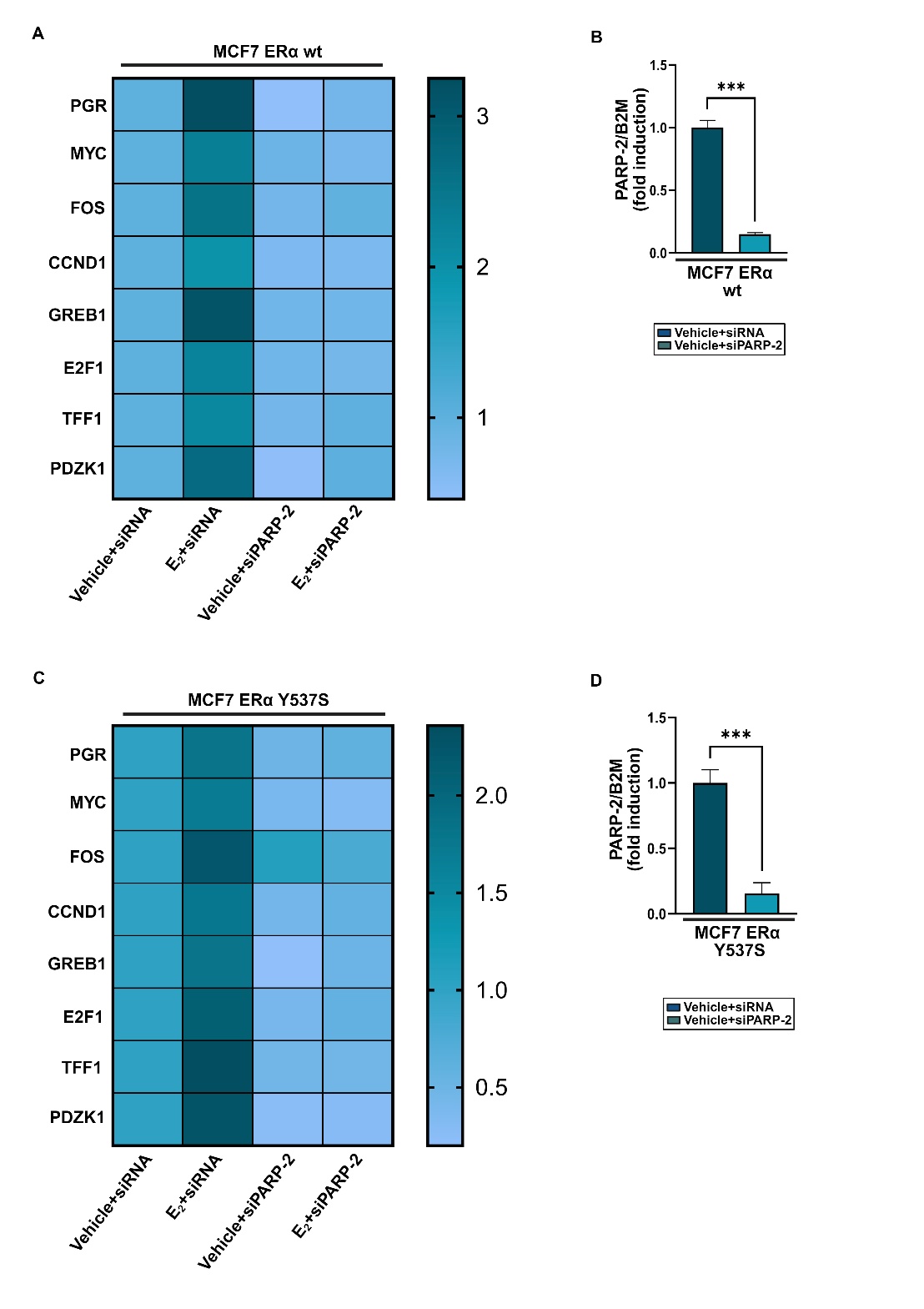
**

**Additional File 2. PARP-2 silencing reduces the expression of main target genes of ERα in breast cancer cells.** mRNA expression of main ERα target genes in ERα wild type (wt) **(A)** and Y537S mutated **(C)** MCF7 cells. Cells were transiently transfected for 36 hours with negative control (siRNA) or siPARP-2 and treated with vehicle or 10 nM 17β-estradiol (E_2_), as indicated. Values are normalized to human beta-2-microglobulin (B2M) endogenous control expression and shown as fold changes of mRNA expression. Efficacy of PARP-2 silencing in ERα wt **(B)** and Y537S mutated **(D)** MCF7 cells. Values are normalized to human beta-2-microglobulin (B2M) endogenous control expression and shown as fold changes of mRNA expression. Data represent the average of three biological replicates with error bars indicating SEM. (***) p < 0.001
