# Supplementary material for: PARP-1 as a novel target in endocrine-resistant breast cancer
Source: J Exp Clin Cancer Res. 2025 Jun 16;44:175. doi: 10.1186/s13046-025-03441-4 (PMC12168341; doi:10.1186/s13046-025-03441-4)
Supplement: Supplementary file 6 — Supplementary Material 6 [file 13046_2025_3441_MOESM6_ESM.docx]

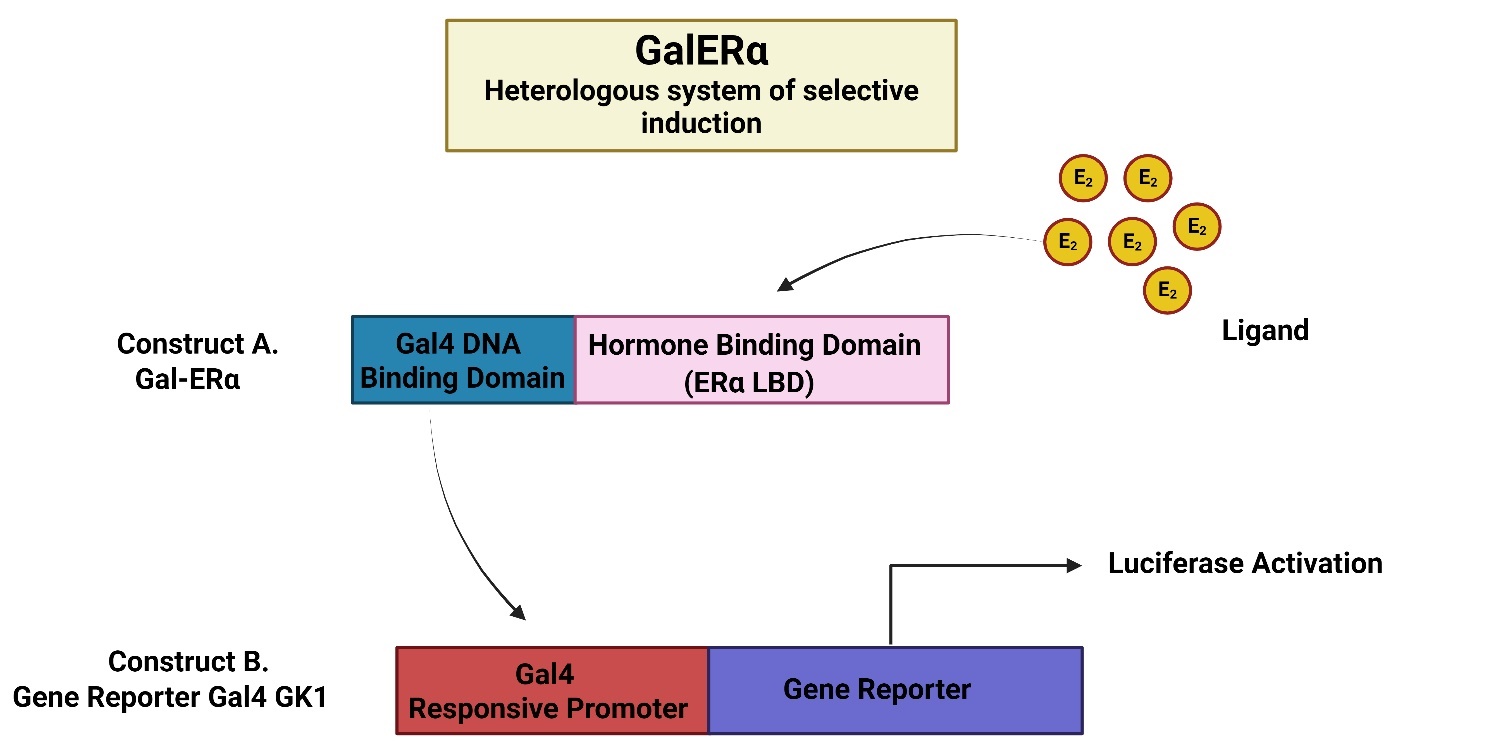


**Additional File 3. GalERα/GK1 plasmid system.** The GalERα System is a heterologous system used to evaluate the activation of the Ligand Binding Doman (LBD) of the Estrogen Receptor α (ERα). The construct A consists of the DNA-binding domain of the yeast Gal4 protein (a positive regulator of gene expression of galactose-induced genes) fused to the hormone-binding domain of the human ERα. The interaction between the LDB and the ligand determines the activation of Gal4 and its recruitment within the promoter of Gal4 (construct B), leading to the regulation of the gene reporter.
