# Supplementary material for: PARP-1 as a novel target in endocrine-resistant breast cancer
Source: J Exp Clin Cancer Res. 2025 Jun 16;44:175. doi: 10.1186/s13046-025-03441-4 (PMC12168341; doi:10.1186/s13046-025-03441-4)
Supplement: Supplementary file 8 — Supplementary Material 8 [file 13046_2025_3441_MOESM8_ESM.docx]

**
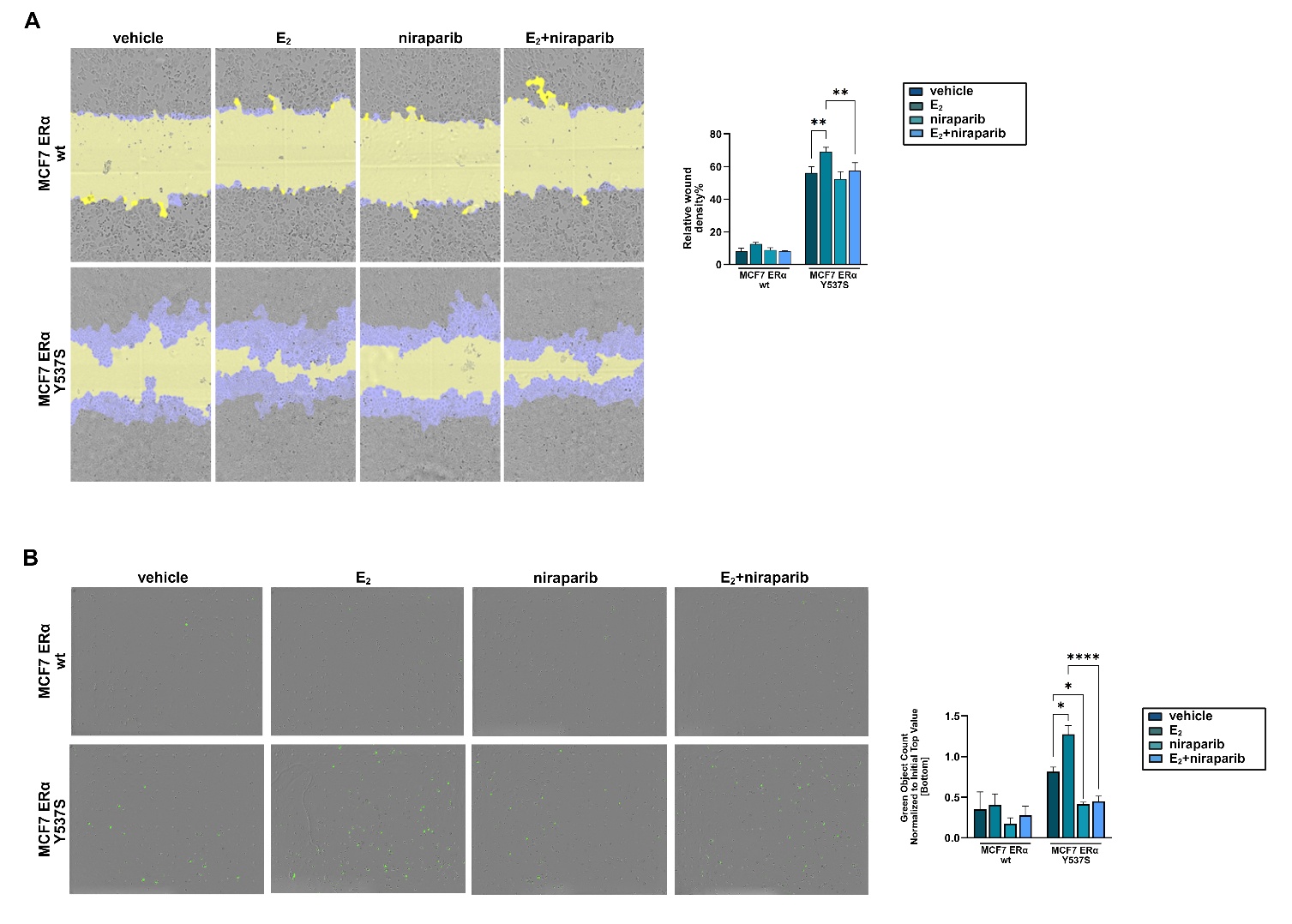
**

**Additional File 5. PARP-1 inhibition prevents the motile potential of ER-positive breast cancer cells.** Scratch Wound Healing Assay **(A)** and invasion assay **(B)** have been respectively used to evaluate, by the Incucyte platform, the migratory and invasive activity ERα wild type (wt) and Y537S mutated MCF7 breast cancer cells treated with vehicle or 10 nM 17β-estradiol (E_2_) alone or in combination with 1μM PARP-1 inhibitor niraparib. Data represent the average of three biological replicates with error bars indicating SEM. (*) p < 0.05; (**) p < 0.005; (****) p < 0.0001.
