# Supplementary material for: PARP-1 as a novel target in endocrine-resistant breast cancer
Source: J Exp Clin Cancer Res. 2025 Jun 16;44:175. doi: 10.1186/s13046-025-03441-4 (PMC12168341; doi:10.1186/s13046-025-03441-4)
Supplement: Supplementary file 9 — Supplementary Material 9 [file 13046_2025_3441_MOESM9_ESM.docx]

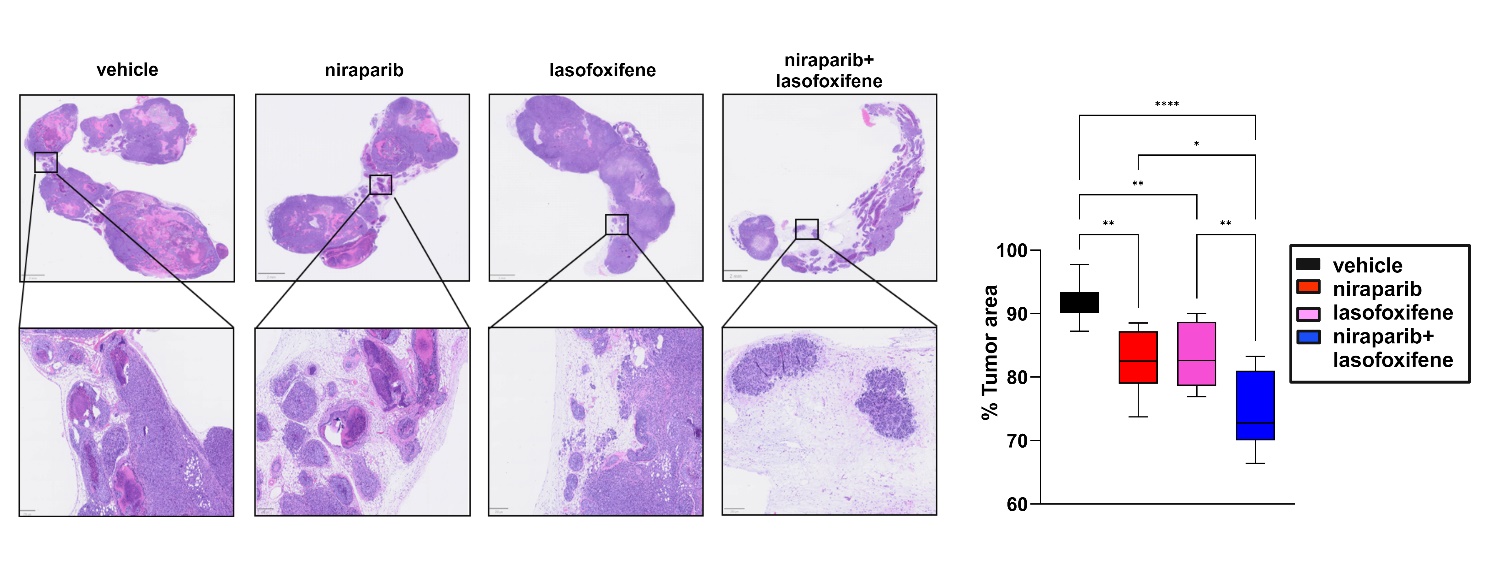


**Additional File 6. Hematoxylin and eosin (H&E) staining of breast cancer sections from mice injected with ERα Y537S mutated MCF7 BC cells.** Representative H&E staining of mammary glands from mice injected with ERα Y537S mutated MCF7 cells treated for 90 days with 10 mg/kg of niraparib and/or 5 mg/kg of lasofoxifene as single treatments or in combination. The right panel represents the percentage of tumor cells found in the breast tumor sections after mice sacrifice. (*) p < 0.05; (**) p < 0.005; (****) p < 0.0001.
