# Supplementary material for: PARP-1 as a novel target in endocrine-resistant breast cancer
Source: J Exp Clin Cancer Res. 2025 Jun 16;44:175. doi: 10.1186/s13046-025-03441-4 (PMC12168341; doi:10.1186/s13046-025-03441-4)
Supplement: Supplementary file 10 — Supplementary Material 10 [file 13046_2025_3441_MOESM10_ESM.docx]

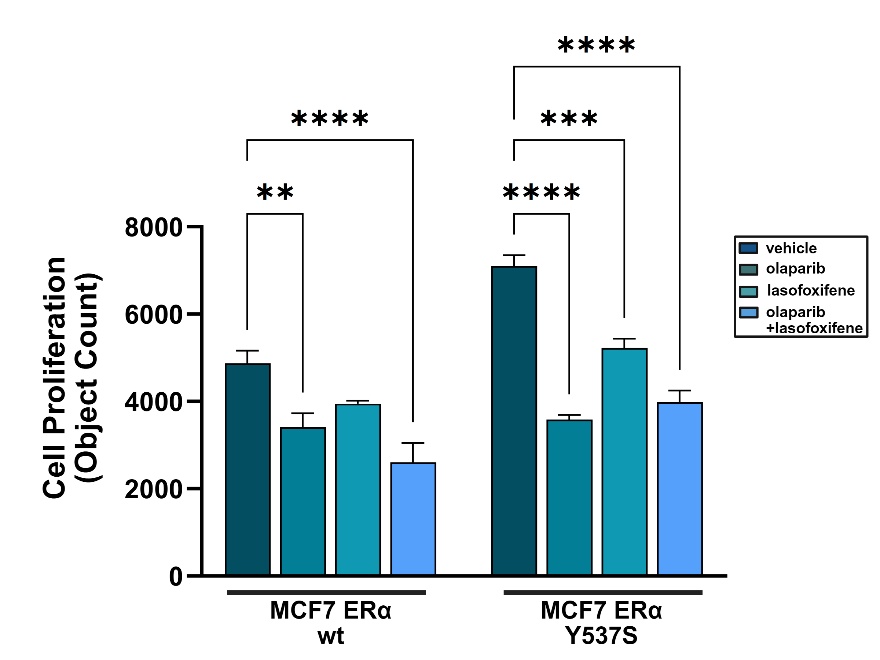


**Additional File 7. Anti-proliferative effects of PARP-1 and ERα inhibition in ERα-positive breast cancer cells.** Proliferation of ERα wild type (wt) and Y537S mutated MCF7 breast cancer (BC) cells after 5 days of treatment with vehicle or 1μM olaparib alone or in combination with 1μM lasofoxifene. Data represent the average of three biological replicates with error bars indicating SEM. (*) p < 0.05; (**) p < 0.005; (****) p < 0.0001.
