# Supplementary material for: PARP-1 as a novel target in endocrine-resistant breast cancer
Source: J Exp Clin Cancer Res. 2025 Jun 16;44:175. doi: 10.1186/s13046-025-03441-4 (PMC12168341; doi:10.1186/s13046-025-03441-4)
Supplement: Supplementary file 11 — Supplementary Material 11 [file 13046_2025_3441_MOESM11_ESM.docx]

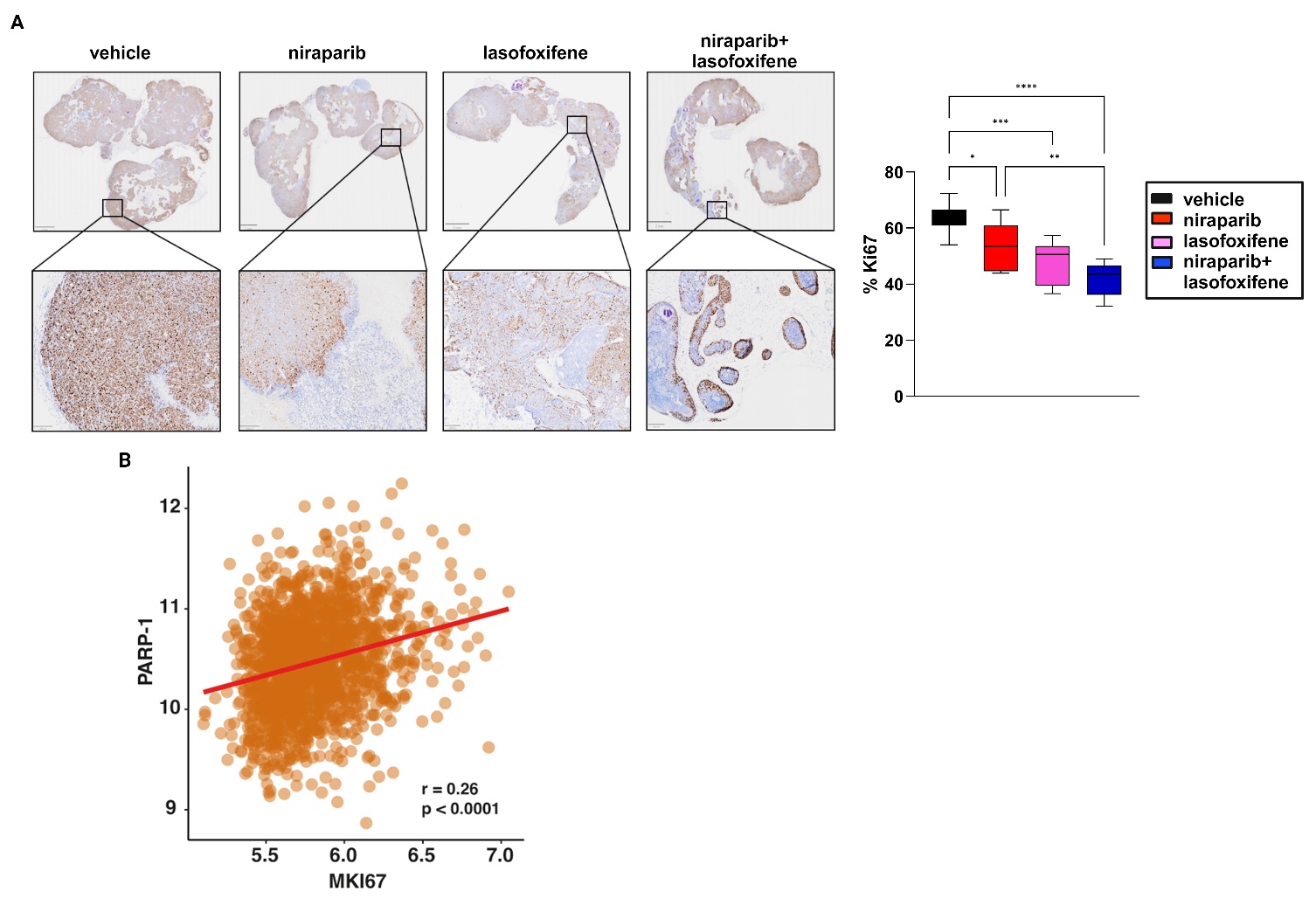
**Additional File 8. Ki67 staining in breast cancer sections from mice injected with ERα Y537S mutated MCF7 cells. (A)** Representative IHC sections of Ki67 staining of mammary glands from mice injected with ERα Y537S mutated MCF7 cells treated for 90 days with 10 mg/kg of niraparib and/or 5 mg/kg of lasofoxifene as single treatments or in combination. Right panel represents the box plot of Ki67% in the mammary glands. **(B)** Scatter plot depicting the significant correlation between PARP-1 and Ki67 (gene name: MKI67) mRNA expression in ER-positive breast cancer patients (n. 1506) of the METABRIC dataset. The Pearson correlation coefficient (r) and the relative p-values are shown in the panel.
